# Counting pseudoalignments to novel splicing events

**DOI:** 10.1093/bioinformatics/btad419

**Published:** 2023-07-11

**Authors:** Luka Borozan, Francisca Rojas Ringeling, Shao-Yen Kao, Elena Nikonova, Pablo Monteagudo-Mesas, Domagoj Matijević, Maria L Spletter, Stefan Canzar

**Affiliations:** Department of Mathematics, Josip Juraj Strossmayer University of Osijek, Osijek 31000, Croatia; Gene Center, Ludwig-Maximilians-Universität München, Munich 81377, Germany; Huck Institutes of the Life Sciences, The Pennsylvania State University, University Park, PA 16802, United States; Biomedical Center, Department of Physiological Chemistry, Ludwig-Maximilians-Universität München, Planegg-Martinsried 82152, Germany; Biomedical Center, Department of Physiological Chemistry, Ludwig-Maximilians-Universität München, Planegg-Martinsried 82152, Germany; Gene Center, Ludwig-Maximilians-Universität München, Munich 81377, Germany; Department of Mathematics, Josip Juraj Strossmayer University of Osijek, Osijek 31000, Croatia; Biomedical Center, Department of Physiological Chemistry, Ludwig-Maximilians-Universität München, Planegg-Martinsried 82152, Germany; School of Science and Engineering, Division of Biological & Biomedical Systems, University of Missouri Kansas City, Kansas City, MO 64110, United States; Gene Center, Ludwig-Maximilians-Universität München, Munich 81377, Germany; Huck Institutes of the Life Sciences, The Pennsylvania State University, University Park, PA 16802, United States; Department of Computer Science and Engineering, The Pennsylvania State University, University Park, PA 16802, United States

## Abstract

**Motivation:**

Alternative splicing (AS) of introns from pre-mRNA produces diverse sets of transcripts across cell types and tissues, but is also dysregulated in many diseases. Alignment-free computational methods have greatly accelerated the quantification of mRNA transcripts from short RNA-seq reads, but they inherently rely on a catalog of known transcripts and might miss novel, disease-specific splicing events. By contrast, alignment of reads to the genome can effectively identify novel exonic segments and introns. Event-based methods then count how many reads align to predefined features. However, an alignment is more expensive to compute and constitutes a bottleneck in many AS analysis methods.

**Results:**

Here, we propose fortuna, a method that guesses novel combinations of annotated splice sites to create transcript fragments. It then pseudoaligns reads to fragments using kallisto and efficiently derives counts of the most elementary splicing units from kallisto’s equivalence classes. These counts can be directly used for AS analysis or summarized to larger units as used by other widely applied methods. In experiments on synthetic and real data, fortuna was around 7× faster than traditional align and count approaches, and was able to analyze almost 300 million reads in just 15 min when using four threads. It mapped reads containing mismatches more accurately across novel junctions and found more reads supporting aberrant splicing events in patients with autism spectrum disorder than existing methods. We further used fortuna to identify novel, tissue-specific splicing events in *Drosophila*.

**Availability and implementation:**

fortuna source code is available at https://github.com/canzarlab/fortuna.

## 1 Introduction

Intronic sequences need to be removed, or spliced, from a transcribed pre-mRNA to produce mature mRNA. Introns can be spliced in alternative ways from the same pre-mRNA to produce distinct combinations of exons in different mRNA transcript variants. This alternative splicing (AS) contributes to the diversity of transcriptomes and proteomes across cell types, tissues, and developmental stages. Aberrant splicing, for example caused by human genomic variants, is prevalent in various diseases, including cancer and neurological disorders (Titus *et al.* 2021). The identification of expressed transcript variants and their roles in normal development and disease is still a field of open investigation. RNA sequencing (RNA-seq) is the preferred technology to study expressed transcript variants.

The quantification of full-length transcripts from short reads produced by RNA-seq is facilitated by the rapid pseudoalignment of reads to known transcripts by methods such as kallisto (Bray *et al.* 2016) and salmon (Patro *et al.* 2017). Instead of performing a computationally expensive alignment between all reads and all transcript sequences, a pseudoalignment simply determines the set of compatible transcripts each read could have originated from. However, pseudoalignment-based analysis as introduced in Bray *et al.* (2016) is defined with respect to known transcripts and will not only miss novel or disease-specific splicing events, but unmappped or mismapped reads from novel junctions can also affect downstream quantification of annotated features.

The differential usage of individual exons, on the other hand, can be detected by methods such as DEXSeq (Anders *et al.* 2012) without making any assumptions about how exons have been combined in potentially novel transcripts. More generally, most event-based methods such as SplAdder (Kahles *et al.* 2016) and rMATS (Shen *et al.* 2014) first summarize reads by counting how many of them support the inclusion of transcript “building blocks” that are assumed indivisible. To identify and quantify potentially novel compositions of such building blocks, reads first need to be aligned to their corresponding sequences. Although such an alignment effectively enables the detection of novel exonic segments and novel splice sites and introns, it imposes a computational bottleneck (Bray *et al.* 2016, Sterne-Weiler *et al.* 2018).

Here, we make use of the fact that a large number of novel introns found in human and other well-annotated species typically do not gap arbitrary stretches of nucleotides in the genome, but rather re-use known splice sites. For example, using recount3 (Wilks *et al.* 2021), a resource that compiles hundreds of thousands of public human and mouse RNA-seq samples, Wilks *et al.* (2021) reported that 31.3% of novel human junctions in the Sequence Read Archive (SRA) combine known splice sites. In only 15.6% of novel junctions, neither donor nor acceptor appeared in the annotation. Among splice junctions that appear in at least 20% of SRA run accessions, almost every second of novel junctions contained exclusively annotated splice sites.

Recognizing the limitation of fast pseudoalignment-based methods to predefined transcript annotations on the one hand, and the high computational cost incurred by more accurate, event-based methods on the other, Whippet (Sterne-Weiler *et al.* 2018) implements a new transcriptome index for fast alignment across novel junctions between annotated splice sites. The index records *k*-mers flanking all combinations of annotated donors and acceptors. This reliance on *k*-mers makes Whippet sensitive to mismatches near splice sites and requires reads across splice junctions to have a minimum overhang of *k*. Furthermore, the heuristic pruning of the search space might eliminate true hits of reads. Connecting all annotated donor and acceptor sites to be able to detect novel junctions has also been proposed by other (alignment-based) methods such as ASGAL (Denti *et al.* 2018). Its high computational cost, however, makes ASGAL suitable for the analysis of single genes or a small set of target genes.

In fortuna, we therefore take a different approach and pseudoalign reads to longer transcript fragments that join splice junctions. A similar idea was adopted by Yanagi (Gunady *et al.* 2019), but it is limited to annotated transcripts. It extracts transcript segments only across annotated splice junctions and can thus not identify novel events. By contrast, we assemble fragments by “guessing” novel introns that combine annotated splice sites in novel, biologically motivated ways. From pseudoalignments we derive counts of the most elementary splicing units which correspond to the number of reads with distinct splicing patterns that were obtained from an extended, well-defined catalog of transcripts. These counts can then be directly used for AS analysis or summarized to larger units such as those used by DEXSeq. In addition, fortuna optionally incorporates novel splice sites identified by STAR (Dobin *et al.* 2013) or any other spliced aligner and annotates all splicing events implied by novel introns.

In experiments on synthetic and real data, we demonstrate a substantial speed advantage of fortuna compared to traditional align and count approaches. We further show that fortuna more accurately maps reads across novel introns in challenging scenarios when reads contain mismatches or span multiple junctions. We use fortuna to catalog thousands of novel splicing events in different *Drosophila* pupal tissues.

## 2 Materials and methods

**Figure 1.**
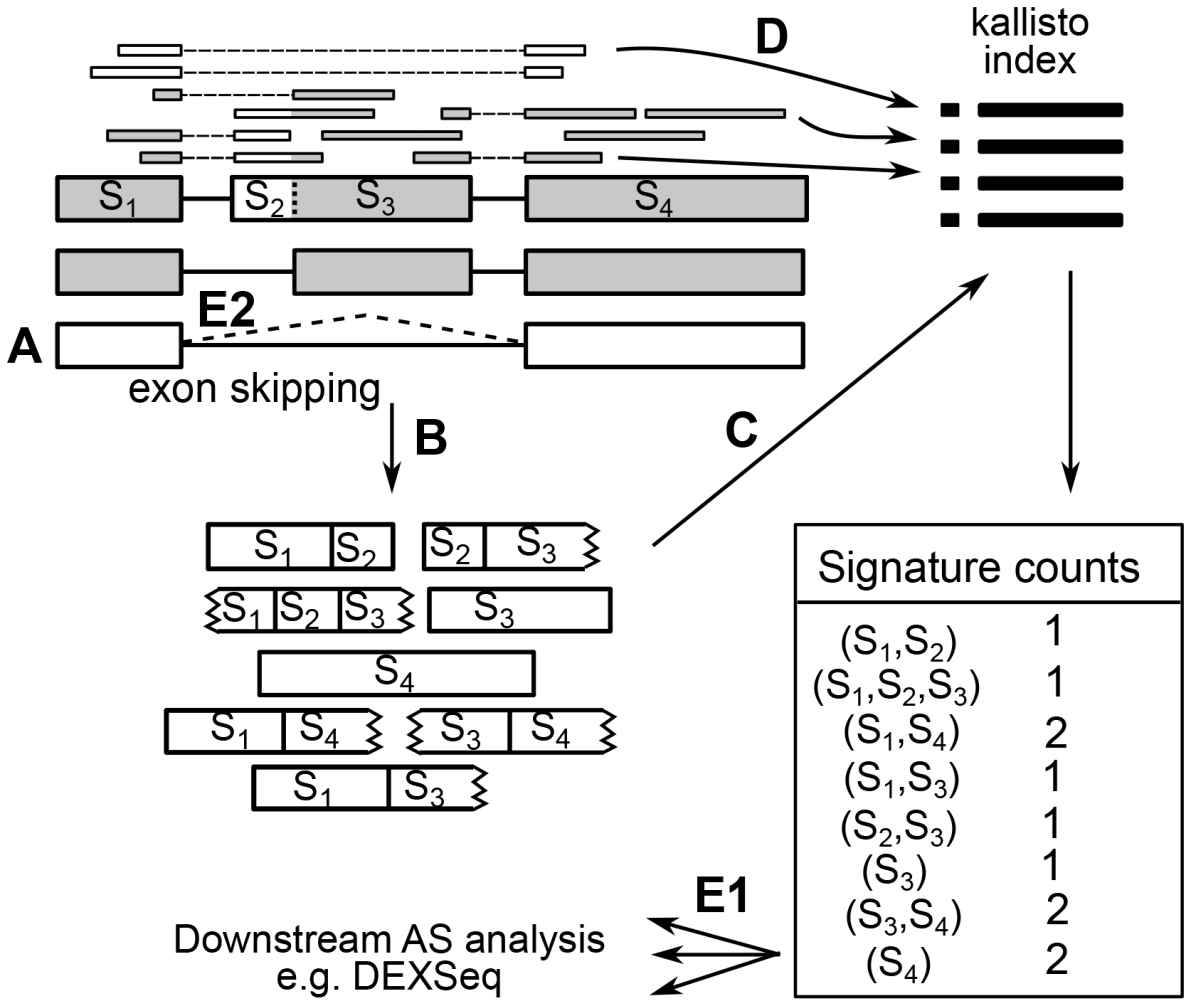
fortuna overview. Detailed description in the main text

The steps that fortuna takes to quantify unambiguous building blocks of transcripts and to detect and annotate novel splicing events are illustrated in Fig. 1. fortuna starts (A) by “guessing” novel transcripts based on annotated splice sites (Section 2.2). It then (B) creates a set of sequence fragments of annotated and guessed novel transcripts that contain all possible combinations of unspliced exonic segments. We design this fragment set to be small with little sequence overlap to reduce required computational resources (described formally in Section 2.3). From this set of fragments we build a kallisto index (C) and use it to efficiently pseudoalign reads to fragments (D), which yields counts of the most elementary splicing units (*signature counts*, introduced in the next Section). Optionally, fortuna can further incorporate novel splice sites (e.g. exonic segment $s_{2}$) identified by any spliced aligner from reads that remained unmapped in step (D), but does not attempt to assemble them to novel exons. Computed counts can be directly used for alternative splicing (AS) analysis or added up to larger units such as those used by DEXSeq (E1). In addition, fortuna annotates all novel events (E2) based on precise definitions of event types (Section 2.4).

### 2.1 Equivalent mapping signatures

**Figure 2.**
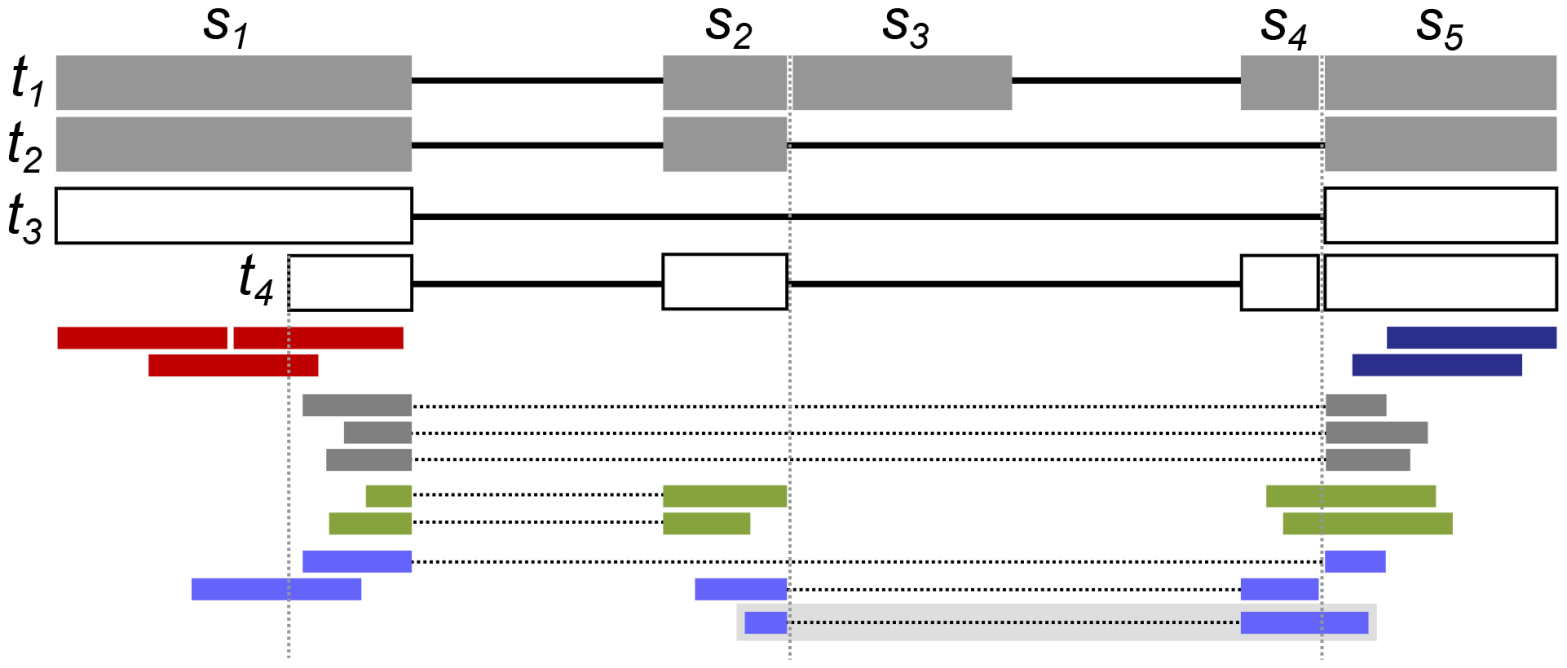
In this example, transcripts $t_{1}$ and $t_{2}$ are assumed to be annotated, while $t_{3}$ and $t_{4}$ are novel. $t_{1}$ and $t_{2}$ partition exons into subexons $s_{1}$–$s_{5}$. Red, dark blue, and spliced green reads are considered pairwise equivalent by kallisto, while Yanagi distinguishes red and dark blue reads in distinct counts. Gray reads from novel junction are ignored in kallisto and Yanagi. DEXSeq counts reads overlapping individual subexons, while Yanagi summarizes red and spliced green reads into a single counting bin. fortuna refines counting bins to mapping signatures, such as (2,4,5) in the case of the shaded blue read

Short reads can be summarized with respect to a catalog of known transcripts by grouping them according to the set of transcripts they could have originated from. kallisto (Bray *et al.* 2016), for instance, considers two reads equivalent if they are compatible with the exact same set of transcripts. The red reads, dark blue reads, and the two spliced green reads in Fig. 2 are compatible with annotated transcripts $t_{1}$ and $t_{2}$ and are thus pairwise equivalent. kallisto then summarizes read data by the sizes of the resulting equivalence classes, often referred to as transcript compatibility counts (TCCs). Reads from the previous example would thus form an equivalence class of size 7. This data summary can serve as input to an EM algorithm to quantify known transcripts (Bray *et al.* 2016), or can be directly compared between conditions (Soneson *et al.* 2016, Cmero *et al.* 2019), or single cells (Ntranos *et al.* 2016).

TCCs by definition rely on known transcripts. Reads obtained from novel transcripts may be ignored, such as the gray reads from novel transcript $t_{3}$ in Fig. 2, or transcripts missing in the annotation may cause mixing of nonequivalent reads, e.g. red and spliced green reads. Another tool Yanagi (Gunady *et al.* 2019) therefore separates alternatively spliced gene regions into distinct counting bins, such that shifts in counts caused by transcripts that combine distant splicing events in novel ways can be detected. For example, an increase in expression of unknown transcript $t_{4}$ in Fig. 2 can yield more reads of the dark blue type but not of the red type, which will be reflected in Yanagi’s distinct counts.

Novel junctions, however, even across annotated splice sites, would still be missed by Yanagi (e.g. gray reads in Fig. 2) and nonequivalent reads may incorrectly be summarized in the same counting bin (red and spliced green reads in Fig. 2). Methods such as DEXSeq (Anders *et al.* 2012) therefore subdivide exons into smaller units that transcripts could combine in novel ways. A set of transcripts naturally partitions exons into the smallest possible segments, or *subexons*, that are bounded by splice sites or transcription start and end sites [introduced previously as, e.g., *segments* (Feng *et al.* 2011) or *blocks* (Beretta *et al.* 2014)]. For example, transcripts $t_{1}$ and $t_{2}$ in Fig. 2 imply subexons $s_{1}$ through $s_{5}$. DEXSeq then counts the overlapping number of aligned reads for every such subexon. Gray reads, for example, contribute a count of 3 to both $s_{1}$ and $s_{5}$. The possible contribution of a single read to counts of multiple subexons could introduce ambiguities in the DEXSeq analysis. Instead, a different approach has been developed to address this problem by considering two reads equivalent if they overlap the exact same sequence of subexons. This preserves as much information as possible about potentially novel connectivity of subexons in transcripts. For instance, the shaded blue read in Fig. 2 overlaps subexons $s_{2}$, $s_{4}$, and $s_{5}$, in this order, and can be generated only by the splicing pattern of $t_{4}$, even though each of the three subexons occur in multiple transcripts. These *(sub)exon paths* were originally introduced in Rossell *et al.* (2014), and have since been used in various splicing analysis methods (e.g. Canzar *et al.* 2016, Alqassem *et al.* 2021).

To more formally state this concept as applied in this work, let a given gene be subdivided into subexons $s_{1},s_{2},\ldots ,s_{n}$, consecutively numbered in increasing order of their genomic coordinates. Then the *mapping signature* of a read *r* denotes the increasing sequence of indices of subexons ${ı}_{r}=(i_{1},i_{2},\ldots )$ read *r* overlaps, such as (2,4,5) in the case of the shaded read in Fig. 2. Then, two reads *r* and $r^{\prime }$ are equivalent if their mapping signatures are identical, i.e. if ${ı}_{r}={ı}_{r^{\prime }}$. We refer to the sizes of the resulting equivalence classes as *signature counts.* Mapping signatures can naturally be extended to paired-end reads. A read pair $r=(r_{1},r_{2})$ has signature $\left({ı}_{r_{1}},{ı}_{r_{2}}\right)$. Two paired-end reads $\left(r_{1},r_{2}\right)$ and $\left(r_{1}^{\prime },r_{2}^{\prime }\right)$ are equivalent if ${ı}_{r_{1}}={ı}_{r_{1}^{\prime }}$ and ${ı}_{r_{2}}={ı}_{r_{2}^{\prime }}$. In the remainder of this work, we restrict the discussion to single-end reads. We describe how fortuna processes paired-end reads in Section 3.4 and discuss the limitation of this approach in Section 4. To simplify notation, we drop the read subscript where the meaning is clear from the context. We similarly model both exons and transcripts as sequences of subexons and denote the sequence of subexon indices that define exon *e* or transcript *t* as ${ı}_{e}$ or ${ı}_{t}$, respectively. We alternatively treat transcripts as sequences of exons and point out the distinction where necessary.

Signature counts computed by fortuna contain sufficient information to allow conversion to other count types without processing raw reads or their alignments, while the reverse is not true. The set of compatible transcripts, and thus the equivalence class of a read as defined by kallisto can be computed from the read’s mapping signature ı as where t(s) denotes the set of transcripts subexon *s* is contained in and $\bar{ı}$ contains subexons that lie between the first and last subexon of ı but are not in ı. Yanagi’s equivalence classes can be obtained by merging overlapping mapping signatures that are compatible with the same set of (annotated) transcripts. In Fig. 2, for instance, red reads [signature (1)] and spliced green reads [signature (1,2)], would be combined into a single counting class, given that transcript $t_{3}$ with its novel junction is missing in the annotation. To generate counts as used in DEXSeq, for every subexon *s* we simply add all counts of mapping signatures that contain *s*. Finally, signature counts can be summed to quantify the elements of splicing graphs. SplAdder, for example, represents subexons and their connections by introns as nodes and edges in a *segment graph*. SplAdder quantifies individual nodes and edges, and thus does not allow reconstruction of mapping signatures which correspond to more complex combinations of elements, e.g. more than one intron, in the segment graph.


$$\underset{i\in ı}{\cap }t(s_{i})\;\setminus \;\underset{i\in \bar{ı}}{\cup }t(s_{i}),$$

### 2.2 Guessing alternative processing of pre-mRNA

Pseudoalignments computed by methods such as kallisto rely on a set of annotated transcripts. Reads are compared to this annotated catalog to determine from which transcript they could have potentially originated. Here, we conceptually extend such an annotated set of transcripts *T* to include additional, undocumented alternative splicing events. For a gene *g*, let $T_{g}$ be the set of transcripts annotated for *g*. In virtual transcriptome $T_{g}^{as}$, we extend $T_{g}$ by transcripts that can be derived from transcripts in $T_{g}$ by alternatively splicing its exons using known donor and acceptor sites. More precisely, $T_{g}^{as}$ additionally contains all transcripts $t^{\prime }$ that can be generated from a transcript $t\in T_{g}$ by skipping exons in *t* and by modifying the boundaries of the remaining exons consistently with donor and acceptor sites contained in other transcripts in $T_{g}$.

For example, novel transcript $t_{3}$ in Fig. 2 is contained in $T_{g}^{as}$, since its novel junction can be obtained by skipping an exon in $t_{2}$. The sequence of introns in $t_{4}$, i.e. its *intron chain*, is also contained in $T_{g}^{as}$, since its junctions either match a junction in $t_{2}$ or combine a donor site in $t_{2}$ with an acceptor in $t_{1}$. Note that transcript $t_{4}$ also uses a novel transcription start site, which we do not attempt to infer in fortuna. More formally, set $T_{g}^{as}$ contains all transcripts that satisfy the following:

Definition 1 (Virtual transcriptome $T_{g}^{as}$). $T_{g}^{as}$  *contains all transcripts* $t^{\prime }$  *such that*

*(a)* *there exists a transcript* $t\in T_{g}$  *such that every exon e of* $t^{\prime }$  *has nonempty overlap with t, and* $t^{\prime }$  *contains the same transcript start site (TSS) and transcription end site (TES) as t*,
*(b)* *no two “touching” exons from different transcripts can be merged [see property (i) of* $T_{g}^{ap}$  *in Supplementary Section S2.1]*,
*(c)* $t^{\prime }$  *contains only annotated donor and acceptor sites [see property (ii) of* $T_{g}^{ap}$  *in Supplementary Section S2.1]*.

Transcripts $t_{3}-t_{6}$ in Supplementary Fig. S1 are examples of novel transcripts created in $T_{g}^{as}$. Supplementary Fig. S2 shows examples of transcripts that are not contained in $T_{g}^{as}$, because they violate property (b) (transcript $t_{3}$) or property (c) (transcript $t_{4}$.). In virtual transcriptome $T_{g}^{ap}$ we mimic more general alternative processing of pre-mRNA, including splicing, 5’ end capping, and 3’ end cleavage. In contrast to $T_{g}^{as}$, annotated splice sites can be combined in novel ways without restriction to the (modified) exons of a reference transcripts and the genomic region bounded by its TSS and TES. The formal definition of virtual transcriptome $T_{g}^{ap}$ can be found in Supplementary Section S2.1. It holds that $T_{g}\subset T_{g}^{as}\subset T_{g}^{ap}$. Transcript $t_{7}$ in Supplementary Fig. S1, for instance, is contained in $T_{g}^{ap}$ but not in $T_{g}^{as}$ since it combines exons from different transcripts.

The guessing of novel transcripts in virtual transcriptomes introduces new combinations of annotated splice sites, but the detection of novel splice sites requires a conventional spliced alignment of reads. fortuna optionally incorporates novel splice sites found by a spliced aligner as described in Supplementary Section S2.3.

### 2.3 Transcript fragments

fortuna never explicitly constructs virtual transcriptomes, but counts mapping signatures that are compatible with fragments extracted from them. Given a read length *l* and a transcriptome *T* (or one of its extensions $T^{as}$ or $T^{ap}$), we define the set of signatures that can have a nonzero count as *feasible signatures*. In other words, a signature ı is feasible if a read of length *l* can be obtained from a transcript in *T*, such that it overlaps exactly subexons in ı. More formally, let $ı\subseteq {ı}^{\prime }$ denote ı to be a subsequence of ${ı}^{\prime }$, and let ı ⊆c ı′ specify a contiguous subsequence ı of ${ı}^{\prime }$. For instance, (1,3)⊆(1,2,3) but $\left(1,3){⊈}_{c}(1,2,3\right)$, while $\left(1,2)\subseteq_{c}(1,2,3\right)$.

Definition 2 (feasible signature). *Let l be the read length and T the set of transcripts. A sequence* ı  *of subexons in T is a feasible signature if and only if it satisfies:*

($f_{1}$) *There must exist a transcript* t∈T  *such that* ı ⊆c ıt.

($f_{2}$) *A read of length l can be sampled from* ı*:* $\sum_{i\in ı}|s_{i}|\geq l.$

($f_{3}$) *If* $ı=(i_{1},\ldots ,i_{m})$  *with* m≥3*, a read of length l must be able to cover all subexons:*

 

$$\sum_{j=2}^{m-1}|s_{i_{j}}|\leq l-2.$$

A read contained in the equivalence class of a feasible signature $ı=(i_{1},\ldots ,i_{m})$ fully covers all subexons $s_{i_{j}}$ with 1<j<m, but may overlap first and last subexons $s_{i_{1}}$ and $s_{i_{m}}$ only partially. More specifically, let $5^{\prime }(s)$ and $3^{\prime }(s)$ denote the genomic coordinate of the first and last nucleotide in subexon *s*, respectively. Then the leftmost nucleotide $5_{l}^{\prime }(ı)$ a read of length *l* can cover in $s_{1}$ and the rightmost nucleotide $3_{l}^{\prime }(ı)$ it can cover in $s_{n}$ are specified by Equations (1) and (2) in Supplementary Section S2.2. We denote by [*i*, *j*] the genomic sequence from coordinate *i* to (including) coordinate *j* and, with a slight abuse of notation, by $\left[s]:=[5^{\prime }(s),3^{\prime }(s)\right]$ the genomic sequence of subexon *s*. Then the minimal nucleotide sequence f(ı) such that every possible read with mapping signature $ı=(i_{1},\ldots ,i_{m})$, for m>1, maps to f(ı) is: where “⋅” denotes the concatenation of nucleotide sequences. For m=1, $f(ı)=[s_{i_{1}}]$. Note that f(ı) is a fragment of a transcript in *T* due to the property $f_{1}$ in Definition 2. Therefore, to determine the mapping signatures of all reads of length *l* obtained from transcripts in *T*, we need to contiguously map them to transcript fragments in the following set *F*:


$$f(ı)=[5_{l}^{\prime }(ı),3^{\prime }(s_{i_{1}})]\cdot [s_{i_{2}}]\cdot \;\ldots \;\cdot [s_{i_{m-1}}]\cdot [5^{\prime }(s_{i_{m}}),3_{l}^{\prime }(ı)],$$

(1) $$F_{1:1}:=\{f(ı)|ı\;\text{is feasible signature given}\;T,l\}$$

A read that maps to a fragment f(ı) in $F_{1:1}$ has mapping signature ı, and $f^{-1}$ is encoded in the fragment name. Set $F_{1:1}$ will contain many fragments that overlap in their sequence, as illustrated in Supplementary Fig. S3. Redundant sequences can be removed by eliminating all signatures that are contained in other signatures, to obtain fragment set $F_{\max}$ (Equation 3, Supplementary Section S2.2). In contrast to $F_{1:1}$, sequences of boundary subexons are included in full in the definition of $F_{\max}$ to include all nucleotide sequences corresponding to contained signatures. Supplementary Section S2.2 contains a description of the algorithm to compute $F_{1:1}$ and $F_{\max}$.

Supplementary Figure S3 demonstrates that $F_{\max}$ can contain a substantially smaller number of fragments than $F_{1:1}$. Since there is no longer a one-to-one correspondence between fragments in $F_{\max}$ and feasible signatures, the precise equivalence class a read *r* belongs to needs to be computed by a linear scan through the subexons the fragment is composed of. Fragments in $F_{\max}$ may mutually overlap in their boundary subexons $s_{i_{1}}$ or $s_{i_{m}}$ that are included in their entire length irrespective of read length (e.g. subexon $s_{7}$ in Supplementary Fig. S3). We therefore reduce sequence overlap by including sufficiently long subexons as singleton fragments which in turn allows us to trim sequences at the end of the remaining fragments to at most l−1 bases in the first and last subexons. This trimming strategy ensures that every mapping that overlaps $s_{i_{1}}$ or $s_{i_{m}}$ also overlaps $s_{i_{2}}$ or $s_{i_{m-1}}$, respectively, and thus does not conflict with mappings to singleton $s_{i_{1}}$ and $s_{i_{m}}$. The trimming of fragments and the final set of fragments *F* generated by fortuna are formally specified in Supplementary Section S2.2.

Theorem 1. *Let* s(f)  *denote the (maximal) feasible signature that gave rise to fragment* f∈F*. Given read length l and transcriptome T, the set of transcript fragments F is correct and complete in the following sense:*

*(1)* *F only contains sequences that can be derived from transcripts in T, namely* ∀f∈F ∃t∈T  *such that* $s(f)\subseteq_{c}{ı}_{t}$.
*(2)* *Any read of length l sampled from a transcript in T can be mapped to some* f∈F.

The proof of Theorem 1 can be found in Supplementary Section S2.2.

### 2.4 Alternative splicing events

fortuna classifies every novel intron implied by a mapping signature as one of the following types of alternative splicing: (Multi-)exon skipping, alternative donor, alternative acceptor, alternative donor-acceptor pair, novel intron in exon [or *exitron* (Wang *et al.* 2021)], and intron retention. Similar to AStalavista (Foissac and Sammeth 2007), we compare a novel intron pairwise to every annotated transcript *t* to identify the implied event type. For formal definitions of events, see Supplementary Section S2.4. Note that the same intron can imply different types of events with respect to different transcripts.

Since fortuna does not attempt to assemble novel exons, it conservatively labels reads as *intron retention* only if they overlap intronic sequences that are not part of any annotated transcript, and within which no further splicing was observed (Supplementary Section S2.4).

**Figure 3.**
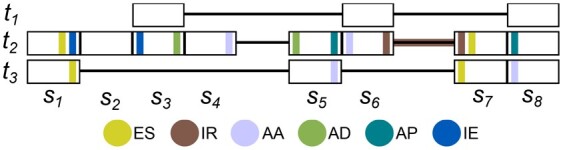
Illustration of splicing event definitions. Transcripts $t_{1},t_{2},t_{3}$ imply subexons $s_{1},\ldots ,s_{8}$. Subexons are colored according to the event type the corresponding (novel) splice junction defines. In this example, mapping signature (1,7) defines a classical exon skipping (ES) with respect to $t_{3}$, and a nonclassical ES wrt $t_{2}$. (3,5) spans an alternative donor (AD) wrt $t_{2}$, (4,6) an alternative acceptor (AA) wrt $t_{2}$, (5,8) an alternative donor-acceptor pair (AP) wrt $t_{2}$ and an alternative acceptor wrt $t_{3}$, (1,3) a novel intron in exon (IE) wrt $t_{2}$, while the subexons $s_{6}$ and $s_{7}$ including the intron between them constitute a novel intron retention (IR)

All reads that do not satisfy the conditions of the above event types are classified as *unknown* by fortuna. Reads with unknown splicing status were either obtained from novel exons or span novel introns that overlap at most one exon in any given transcript. Both cases would require an assembly of reads to be correctly classified. Figure 3 (and Supplementary Fig. S4) illustrates all event types.

## 3 Results

### 3.1 fortuna more accurately detects novel splice junctions than existing methods

In this section we evaluate the accuracy of fortuna and alternative methods in identifying novel junctions between annotated splice sites. We used FluxSimulator (Griebel *et al.* 2012) to generate two simulated data sets containing 80 million reads of length 75 and 100 bp. Reads were obtained from human transcripts annotated in NCBI’s RefSeq database (release 109) and included sequencing errors according to an Illumina error profile predefined by FluxSimulator. To mimic an incomplete catalogue of known splice junctions, we used AStalavista (Foissac and Sammeth 2007) to identify exon skipping events and alternative acceptor and donor sites in our simulated data sets and subsequently removed all transcripts that supported such an event type, i.e. that contained the corresponding intron. Even though fortuna introduces more complex splicing alterations in its virtual transcriptomes, for ease of interpretability we focus the analysis on these simple, most abundant types of splicing events (Sammeth *et al.* 2008) that involve a single alternative splice site.

**Figure 4.**
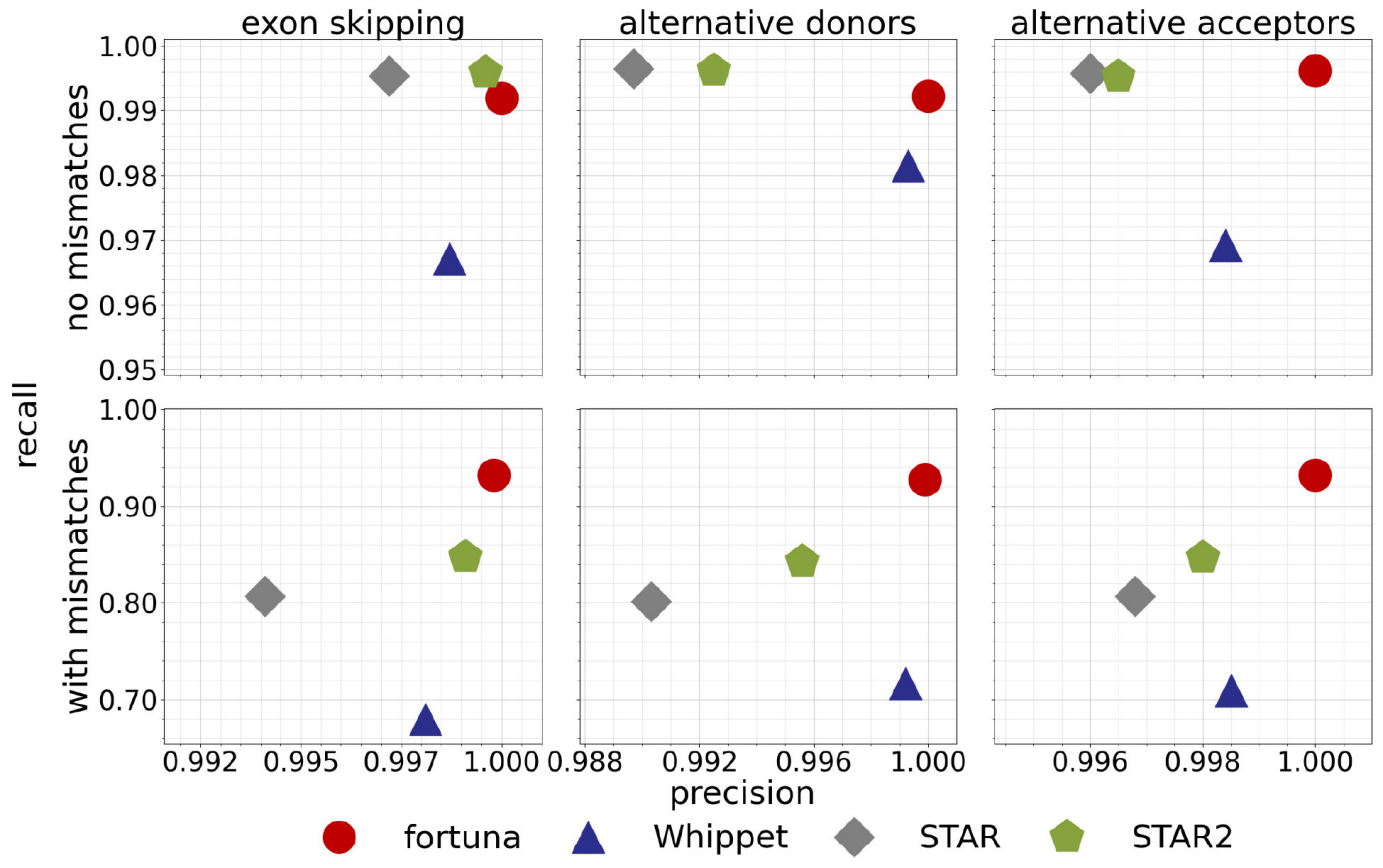
Precision and recall in finding novel junctions between annotated splice sites. Results of fortuna, Whippet, STAR and STAR with two-pass mode (STAR2) are shown for the simulated dataset with 75 bp reads. Reads were split into error-free reads (upper row) and reads containing mismatches (bottom row). Results are stratified by event type (columns)

We then provided each method with the full read data, but only the partial transcript annotation. We benchmarked all methods with respect to simulated reads spanning novel junctions that re-combine annotated donor and acceptor sites. In both datasets (75 and 100 bp), we split this set of reads into error-free reads (234,432 and 302,409) and reads that contained at least one mismatch (75,594 and 141,213). Consistent with Srivastava *et al.* (2016), we consider a read a true positive if at least one of its alignments computed by a method matches its true origin across a novel junction. Accordingly, if none of its alignments matches its true origin, we define the read to be a false positive. We counted unmapped reads as false negatives. Based on these definitions, we report in Fig. 4 recall and precision on the 75-bp dataset comparing fortuna to methods Whippet and STAR, which are both able to map reads to novel junctions. STAR is a general purpose spliced aligner whose output is typically utilized by specialized AS analysis methods such as LeafCutter (Li *et al.* 2018), rMATS (Shen *et al.* 2014), or SplAdder (Kahles *et al.* 2016). We ran STAR once in 1-pass and once in 2-pass mode using default options, and provided the same partial annotation during index generation as to all other methods. We let fortuna guess transcripts according to $T^{as}$ (see Definition 1). As expected, STAR run in 2-pass mode achieved slightly better results than its faster 1-pass variant. For error-free reads, STAR benefited from the second run mostly in terms of precision. When reads contained mismatches, the second STAR run also improved sensitivity. fortuna consistently detected novel junctions with the highest precision, with only a minor drop in sensitivity when inferring novel exons skipping or alternative donors from error-free reads. For reads containing mismatches, fortuna paired the highest sensitivity with the highest precision across event types. Whippet, on the other hand, detected the fewest reads across novel junctions, and mismatches caused its sensitivity to drop further to between 68% and 71%. A similar pattern can be observed for 100 bp long reads (Supplementary Fig. S5). Taking junction reads with and without errors together, fortuna consistently achieved highest recall and precision (Supplementary Table S1). The number of hits per read for STAR, Whippet, and fortuna were 1.02, 1.04, and 1.16, respectively, with a marginally higher value obtained by STAR in 2-pass mode than in 1-pass mode. Even when considering only annotated junctions, their explicit construction by fortuna facilitates the mapping of reads across them (Supplementary Fig. S6, Supplementary Table S2). As expected, in this setting the two-pass mode does not provide any advantage for STAR. We have also compared fortuna to ASGAL (Denti *et al.* 2018), a spliced aligner that is able to detect novel splicing events. Consistent with other methods in this benchmark, we evaluated the alignments across novel junctions produced by ASGAL and ignored the subsequent event identification step. ASGAL aligns reads with a competitive precision but achieves substantially smaller recall values, especially when reads contain mismatches (Supplementary Table S1). For genome-wide analysis, ASGAL relies on Salmon (Patro *et al.* 2017) to split input reads into one instance per gene. This might leave some reads from novel junctions unmapped (Denti *et al.* 2018) potentially causing a drop in recall. Due to recall values that lie far outside of the shown range, we did not include ASGAL in Fig. 4 and Supplementary Fig. S5. ASGAL was designed to run on a single gene or a small set of target genes. For the genome-wide analysis on the two simulated datasets, ASGAL took more than 32 h. Because of its excessive running time and its different intended use we exclude it from the following experiments, which involve more than three times larger datasets.

### 3.2 fortuna finds more reads supporting aberrant splicing events in patients with autism spectrum disorder

Noncoding genetic variants that disrupt mRNA splicing play an important role in rare genetic diseases (Cooper *et al.* 2009). In Jaganathan *et al.* (2019), the authors identified a set of *de novo* mutations that they predicted to alter splicing in individuals with intellectual disability or autism spectrum disorder (ASD). The authors validated 21 aberrant splicing events associated with the predicted *de novo* mutations based on RNA-seq reads spanning the affected splice junction(s). Here we use fortuna to re-align reads from RNA-seq experiments performed in Jaganathan *et al.* (2019) on peripheral blood-derived lymphoblastoid cell lines from individuals with ASD. We compared the number of reads supporting the aberrant splicing events found by fortuna to the original study as well as to the number of reads found by STAR and Whippet. STAR was run with default options and was provided the same transcript annotation (RefSeq, release 109) during index generation as fortuna and Whippet. fortuna was run to guess novel transcripts according to the definition of transcriptome $T^{as}$ (Definition 1 in Section 2.2). In fortuna we only retained the single alignment designated as primary by kallisto. The eight aberrant exon skipping events reported in Jaganathan *et al.* (2019) correspond to the eight RNA-seq samples listed in Supplementary Table S3. We excluded sample 28, as the exon skipping event in this sample is contained in the RefSeq annotation used in our experiments. Across exon skipping events, the three methods agree on a large fraction of junction spanning reads (Supplementary Fig. S7). The original publication (Jaganathan *et al.* 2019) used OLego (Wu *et al.* 2013) to align reads. Jaganathan *et al.* (2019) tried to eliminate OLego’s reference bias caused by its dependence on splicing motifs, but overall fewer supporting reads were found possibly due to disrupted motifs (Supplementary Table S3). On the skipping event in sample 20, STAR and fortuna agreed on a large number of reads (196) which remained unmapped by Whippet. Across events, fortuna detected the largest number of supporting reads, with a substantial number of reads uniquely found by fortuna in all but two samples. Conversely, each read mapped by STAR across one of the aberrantly spliced novel junctions was identically mapped by fortuna. Visual inspection of reads mapped uniquely by fortuna revealed that all but 35 reads in sample 36 spanned at least two introns, which makes them particularly challenging to identify. The remaining 35 reads were mapped uniquely by fortuna across the aberrantly spliced intron reported in Jaganathan *et al.* (2019). This shows that fortuna has a higher sensitivity to detect difficult AS events, and outperforms other analysis tools.

The eight samples listed in Supplementary Table S4 contained novel acceptor or donor sites that fortuna detected using STAR in a second phase to align reads that were not mapped to annotated transcripts or novel donor and acceptor combinations guessed by fortuna (option—refine, see Supplementary Section S2.3). As expected, fortuna and STAR agreed on the exact set of supporting reads, while Whippet was not able to identify any such read (Supplementary Table S4). To be able to detect novel splice sites, Whippet needs to be provided with the (full) alignments of reads previously computed by a conventional read mapper such as STAR, which, in contrast to fortuna, eliminates its original speed advantage over traditional alignment methods. Compared to STAR alignments, only ∼2% of novel junctions between annotated splice sites were missed by fortuna (Supplementary Table S5), which demonstrates the effectiveness of its underlying virtual transcriptome.

### 3.3 fortuna aligns and counts faster than existing methods

To examine the running time of fortuna as a function of the number of reads, we randomly sampled between 10% and 90% of reads in ASD sample 29, which contained ∼291 million 151 bp paired-end reads. CPU times were measured on a 2.30 GHz Intel^®^ Xeon^®^ E5-2697 v4 processors with 320 GB memory. STAR does not count reads in genomic features, and thus needs to be combined with counting methods such as SplAdder, sigcount (Canzar *et al.* 2016), htseq-count (Anders *et al.* 2015), or featureCounts (Liao *et al.* 2014). The latter two methods count reads that overlap any of a set of genomic intervals and thus cannot count reads that exactly overlap all intervals (here subexons) of a mapping signature. We therefore provide the total time required to run STAR (in faster 1-pass mode) combined with either SplAdder or sigcount, with a prior sorting and indexing of alignments using samtools (Li *et al.* 2009). sigcount is based on the exact same definition of signature counts and uses efficient algorithms and data structures implemented in the SeqAn library (Döring *et al.* 2008). SplAdder quantifies reads in (sub)exons and across introns but cannot distinguish more complex combinations of subexons. We included SplAdder in this benchmark since it was used in a large-scale study of alternative splicing in almost 9000 tumor samples (Kahles *et al.* 2018), and since signature counts used by fortuna can be converted in principle to exon/intron counts as used in SplAdder. We ran SplAdder with option –quantify-graph to omit the extraction of AS events. Consistent with previous experiments, fortuna was run to guess novel transcripts in $T^{as}$ (Definition 1).

**Figure 5.**
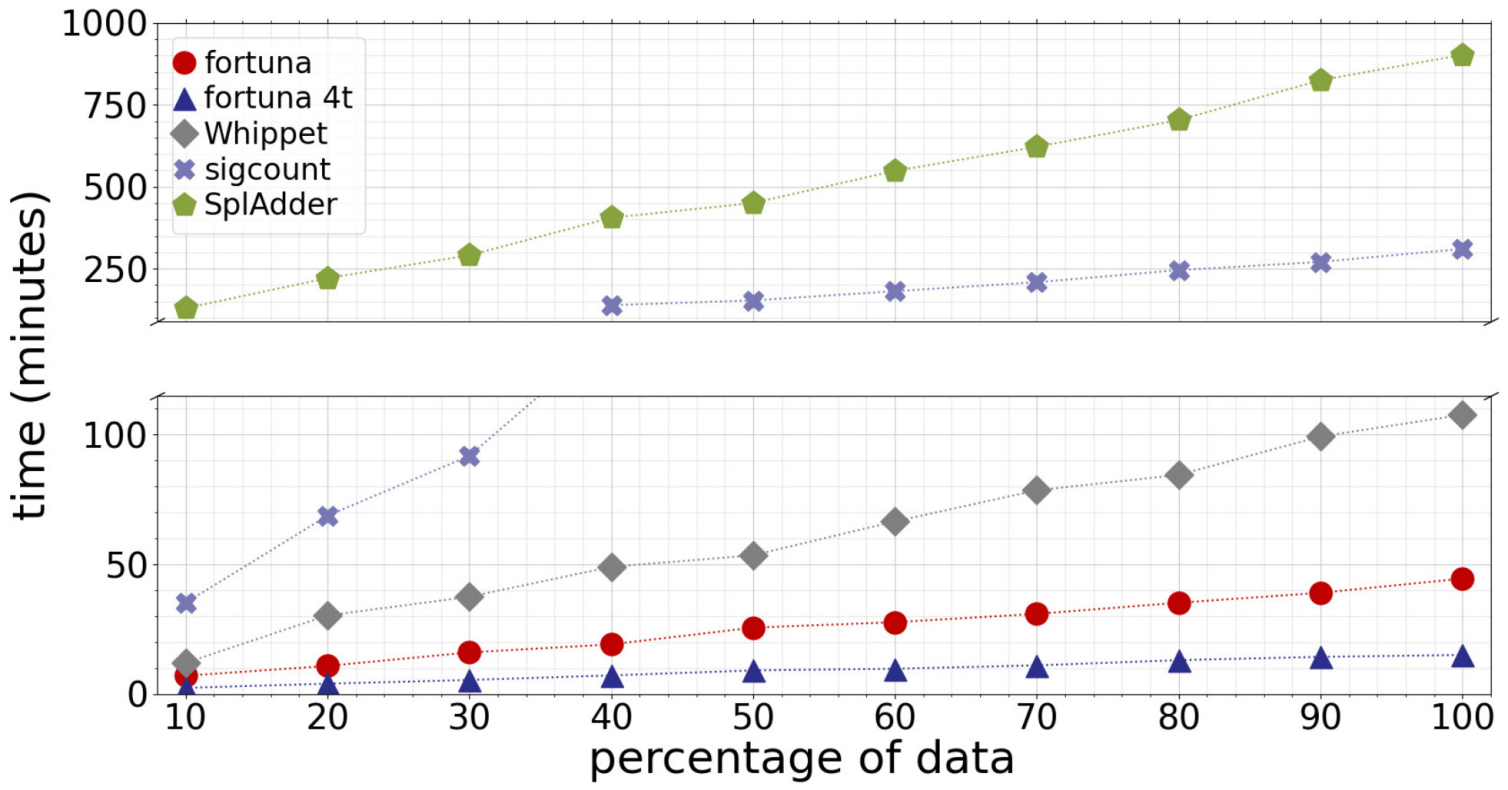
Running time in minutes of fortuna and competing methods on random subsamples of an ASD sample with 291 million reads

Figure 5 demonstrates a clear speed advantage of alignment-free methods Whippet and fortuna compared to conventional align & count approaches. fortuna is more than two times faster than Whippet on all but the smallest sample size. In contrast to Whippet, fortuna can further benefit from multiple threads and was able to count 291 million reads with equivalent mapping signatures in around 15 min using just four threads. With the same number of threads, STAR (1-pass) combined with sigcount took 159 min and was thus still slower than Whippet using a single thread.

Supplementary Table S6 lists the total running times of all methods, which are further split into times required for alignment, sorting, and counting in Supplementary Table S7. As expected, running STAR in the more accurate 2-pass mode roughly doubled the time required for the alignment step. Note that fortuna and Whippet circumvent writing the (pseudo)alignments of reads to disk which can be time-consuming. In Supplementary Table S8, we report running times of fortuna and Whippet when making use of the option to output aligned reads in addition to counts. Peak memory usage was smallest for methods Whippet (2 GB) and kallisto (4 GB). fortuna used up to 24 GB of memory, compared to 29 GB for STAR. For a shorter read length of 75 bp as used in one of our simulations, fortuna’s peak memory usage decreased to below 10 GB.

In addition, we ran STAR on reads that fortuna was not able to map to transcripts in $T^{as}$ which we then used to refine signature counts (option –refine, see Supplementary Section S2.3). After providing fortuna with 291 million reads, STAR was run on ∼20 million unmapped reads, ∼3 million of which it successfully aligned in 25 min (single-threaded). The final partitioning of subexons and the adjustment of signature counts by fortuna took <2 min. This shows that fortuna can flexibly incorporate novel splice sites without sacrificing its core speed advantage.

Finally, we show in Supplementary Table S9 that the set of fragments *F* (equation (4), Supplementary Section S2.2) fortuna constructs is indeed smaller than $F_{1:1}$ (1) and contains less sequence redundancy than $F_{\max}$ (equation (3), Supplementary Section S2.2), and thus yields the best trade-off between running time and memory usage.

### 3.4 fortuna identifies novel, tissue-specific events in *Drosophila*

**Figure 6.**
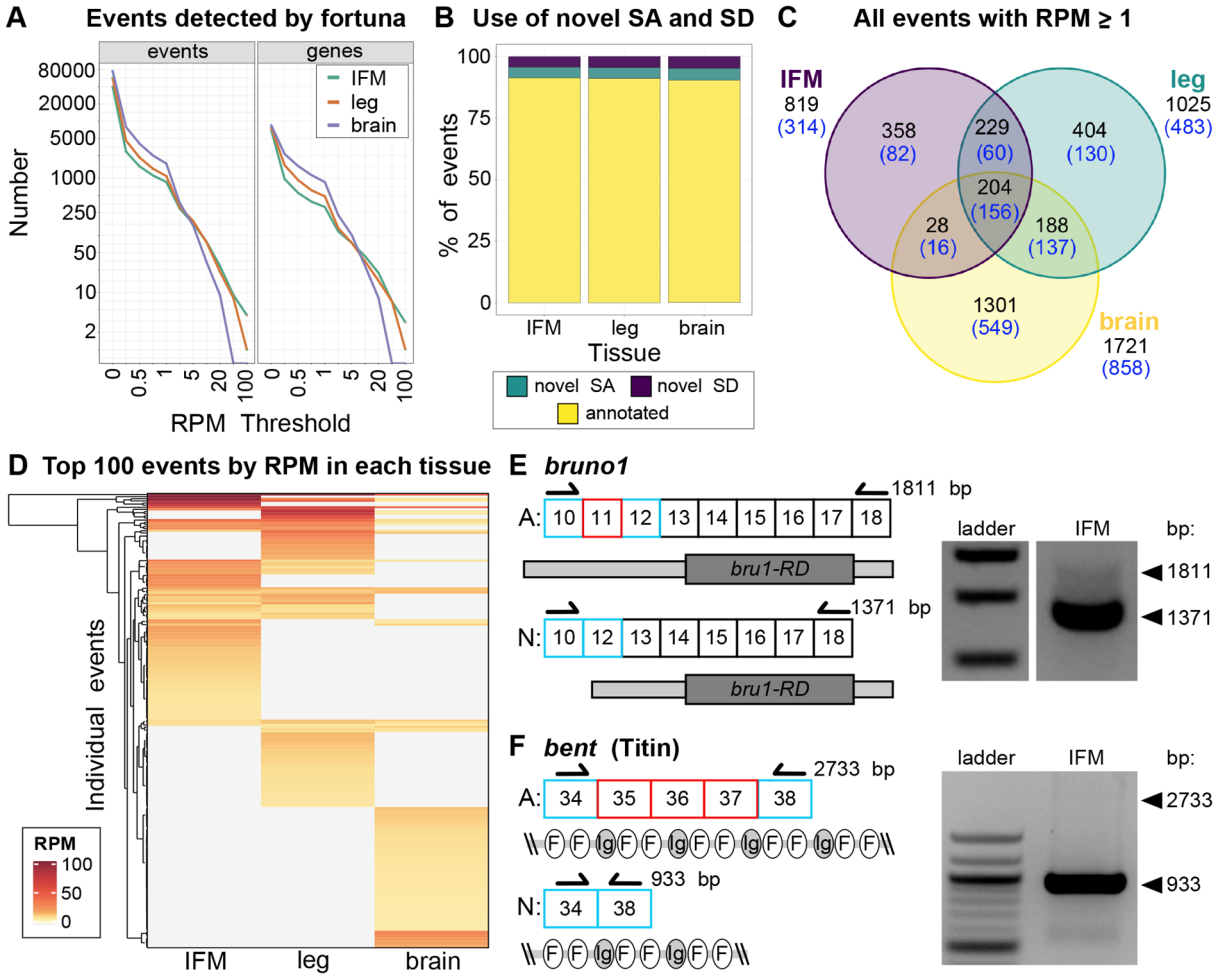
fortuna detects novel, tissue-specific events in *Drosophila*. (A) Line plot showing the number of novel events (left) or genes containing novel events (right) in indirect flight muscle (IFM, green), leg (orange) and brain (purple) samples dissected from *Drosophila* at 72 h after puparium formation. Samples were evaluated at various RPM thresholds. (B) Bar plot of the percent of events (RPM ≥ 0) utilizing a novel splice acceptor (SA, cyan), a novel splice donor (SD, purple) or annotated SA/SD (yellow). (C) Venn diagram of the overlap in novel events (top, black numbers) and genes containing events (bottom, blue numbers) between IFM (left circle, purple), leg (right circle, cyan) and brain (bottom circle, yellow) at RPM ≥ 1. (D) Clustering and heatmap of event RPM for the top 100 events in all three tissues. RT-PCR on IFM confirming novel events in *bruno1* (*bru1*) (E) and *bent* (*bt*, Titin) (F). Annotated (A) and novel (N) isoform lengths in basepairs (bp), as well as exons joined by the novel events (cyan, left most boxes in N), skipped exons (red, present in A but absent in N) and primers (arrows) are illustrated. The *bru1* event results in a shorter 5’-UTR on the *bru1-RD* mRNA isoform (coding: dark gray box, UTR: light gray boxes). The event in *bt* produces a shorter Projectin protein isoform lacking several Fibronectin-3 (FN3, F) and Immunoglobulin (Ig) domain repeats

We next demonstrate the application of fortuna to gain novel, biologically relevant insight into tissue-specific splicing in the model organism *Drosophila*. We tested fortuna on paired-end RNA-seq data generated from different *Drosophila* pupal tissues, including dissected brain, indirect flight muscle (IFM) and whole leg. fortuna (pseudo-)aligned the two reads of each pair independently and reported the sum of counts over both ends. fortuna identified thousands of novel splicing events mapping to hundreds of genes in each tissue, even after stringent filtering (Fig. 6A). About 10% of these events utilize novel splice acceptors or donors, while the majority reflect novel junctions between annotated splice sites (Fig. 6B). Although we observed novel instances of all types of splice events, exon skipping events were most prevalent (Supplementary Fig. S8A). Only ∼2% of novel events are supported by 150 or more reads (∼RPM > 1), while more than 60% are supported by five or fewer reads (Supplementary Fig. S8B, Supplementary Table S10), likely reflecting noise in the data given the sequencing depth. Thus, subsequent analysis was performed on events with an RPM ≥ 1, to focus on the most biologically relevant events. We observed only weak correlation between event RPM and gene length, locus complexity or expression level at any filter level (Supplementary Fig. S8C–E; Supplementary Table S10).

We hypothesized that the novel events might reflect tissue-specific splicing that is currently unannotated. Indeed, we found that only 7.5% of events with an RPM ≥ 1 are shared across all three tissues, while 13%, 15%, and 48% are IFM, leg and brain specific, respectively (Fig. 6C). This is also reflected in a heatmap of the top 100 events in each tissue (Fig. 6D). Many events are tissue-specific or show dramatically different RPM levels between tissues. The genes harboring these novel events are diverse, and also reflect underlying tissue-specific functionalization as revealed by gene ontology enrichments (Supplementary Fig. S9A and B). For example, while genes harboring shared events are enriched in common biological process terms such as “cytoskeleton organization” and “behavior,” brain-specific genes are enriched in “synapse organization,” “neuron recognition,” and “ion transport” reflecting neuronal-specific processes. By contrast, IFM and leg-specific genes are enriched in “actomyosin structure organization,” “oxidation-reduction process,” “flight,” and “mesoderm development,” reflecting muscle-specific processes.

We also noted that the number of novel events per gene is tissue-specific and moderately correlated with locus expression level (Supplementary Fig. S8D and E; Supplementary Table S10). Many novel events are found in genes that undergo tissue-specific alternative splicing, for example genes that encode essential sarcomere proteins such as *Mhc*, *bt*, *Unc-89*, *up*, *Tm1*, *wupA*, *Strn-Mlck* and *sls* all have more than 10 novel events in IFM or leg samples (Supplementary Fig. S9C, Supplementary Table S10). By contrast, in brain, genes relevant for neuronal activity such as *kcc*, *slo*, *Atp*α, *CaMKII*, *brp*, *Cadps*, *stj*, and *Rdl* have more than 10 novel events. We verified several novel events in IFM using RT-PCR. One novel exon skipping event in *bru1*, an essential regulator of IFM-specific splicing, results in a shorter 5’-UTR structure of the *bru1-RD* isoform mRNA (Fig. 6E). Another (multi) exon skipping event we confirmed in *bt*, which encodes Projectin, a Titin isoform that forms connecting filaments in the sarcomere, results in attenuation of a protein region rich in flexible Fibronectin-3 (FN3) and immunoglobulin (Ig) domains (Fig. 6F) that may impact sarcomere stiffness. These findings illustrate the utility of fortuna to identify novel, biologically relevant alternative splice events. Moreover, this data illustrates the importance of including novel splice event discovery in RNA-seq analysis pipelines, even for “wildtype” samples, as tissue or temporal specific splicing events may not be adequately represented in existing transcriptome annotations.

## 4 Conclusion

We have introduced fortuna, a novel method that allows quantification of novel splicing patterns using fast pseudoalignments. It is several times faster than conventional align and count methods and its explicit construction of fragments containing guessed splice junctions facilitates the identification of novel introns in challenging scenarios.

fortuna counts reads that are equivalent in terms of their splicing of unambiguous units that do not contain any splice sites. These signature counts can be used as input by various AS analysis methods, and are used by fortuna to infer and annotate novel introns. Furthermore, any counting unit based on transcriptomic features alone can be reconstructed from signature counts. We provide a script that within seconds converts signature counts to counts as defined and used by DEXSeq.

fortuna currently does not take into account the pairing information of reads during mapping. It computes the signature of each read independently and optionally allows to combine them subsequently to more general signatures and counts of paired-end reads (see Section 2.1). In our experiments on paired-end read data (Section 3.4), we were interested in the total number of individual reads supporting novel junctions, regardless of read pairing. For other types of downstream analysis, signature counts of paired-end reads might contain valuable information that fortuna is able to reconstruct from signatures of individual reads. Using pairing information during mapping could potentially further improve mapping accuracy, but in contrast to genomic mappings the unknown structure of the originating transcript would only impose weak constraints on mapping locations.

Even though Whippet previously accelerated AS analysis compared to classical alignment-based approaches, fortuna is more than two times faster than Whippet and can further benefit from multiple threads. More importantly, our experiments show that Whippet’s increased speed comes at the cost of a lower sensitivity, especially when mismatches affect flanking *k*-mers. In addition, Whippet will miss reads with an overhang smaller than *k* and can include novel splice sites only when additionally running a spliced aligner on the entire dataset.

We also used fortuna to explore novel splice junctions in *Drosophila*, demonstrating the broad applicability of our tool across organisms. We identified hundreds of novel AS events reflecting distinct, tissue-specific expression signatures. fortuna’s speed and sensitivity thus make it possible to combine novel junction discovery with other RNA-seq analysis pipelines to supplement gaps in existing annotations and to obtain a more comprehensive and biologically relevant catalog of AS events between cells and tissues in normal development and in neuromuscular disease.

## Supplementary Material

btad419_Supplementary_Data

## Data Availability

All mRNA-Seq data are publicly available from GEO under accession number GSE194199.
