# Supplementary material for: Counting pseudoalignments to novel splicing events
Source: Bioinformatics. 2023 Jul 11;39(7):btad419. doi: 10.1093/bioinformatics/btad419 (PMC10348833; doi:10.1093/bioinformatics/btad419)
Supplement: btad419_Supplementary_Data [file btad419_supplementary_data.pdf]

Supplementary Material
Counting pseudoalignments to novel splicing events

Luka Borozan[1], Francisca Rojas Ringeling[2], Shao-Yen Kao[3], Elena Nikonova[3], Pablo Monteagudo-Mesas[4], Domagoj Matijević[1], Maria L. Spletter*[3,5], and Stefan Canzar†[6,7]

[1]Department of Mathematics, University J. J. Strossmayer in Osijek, Osijek, Croatia
[2]Department of Chemistry, The Pennsylvania State University, University Park, PA, USA
[3]Biomedical Center, Department of Physiological Chemistry, Ludwig-Maximilians-Universität München, Martinsried-Planegg, Germany
[4]Gene Center, Ludwig-Maximilians-Universität München, Munich, Germany
[5]School of Science and Engineering, Division of Biological & Biomedical Systems, University of Missouri Kansas City, MO, USA
[6]Department of Computer Science and Engineering, The Pennsylvania State University, University Park, PA, USA
[7]Huck Institutes of the Life Sciences, The Pennsylvania State University, University Park, PA, USA

*maria.spletter@umkc.edu
†canzar@psu.edu

1 Supplementary Figures and Tables

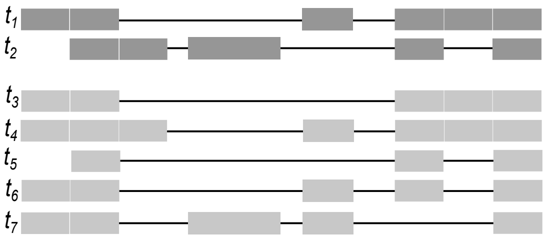

Figure S1: Examples of alternatively spliced and processed transcripts added in T_g^{as} and T_g^{ap}. Assuming transcripts t_1 and t_2 are annotated in T_g, t_3 is generated in T_g^{as} by skipping an exon in t_1, t_4 by using an alternative donor site in t_1, t_5 by skipping an exon in t_2 and using an alternative donor site, and t_6 by excising an intron in t_1 found in t_2. Transcript t_7 is an example of an alternatively processed transcript in T_g^{ap} that is not in T_g^{as}. The second exon of t_7 does not overlap t_1, its third exon does not overlap t_2. t_7 thus violates property (a) in Definition 1.

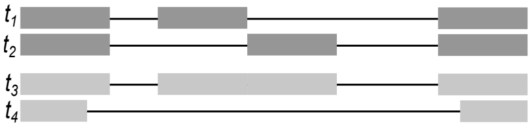

Figure S2: Examples of transcripts that are not contained in T_g^{as} and T_g^{ap}. Assuming transcripts t_1 and t_2 are annotated in T_g, t_3 merges "touching" exons from t_1 and t_2 and thus violates property (i) of T_g^{ap} (Supplementary Section 2.1) and property (b) of T_g^{as} (Definition 1). Transcript t_4 contains a novel splice sites and thus violates property (c) of T_g^{as} and property (ii) of T_g^{ap}.

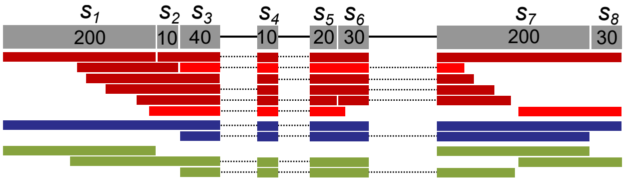

Figure S3: Fragments contained in $F_{1:1}$ (light and dark red), F_{max} (blue) and F as constructed by fortuna (green) from a transcript consisting of 8 subexons. Read length is assumed to be 100bp, lengths of the subexons in bp as shown.

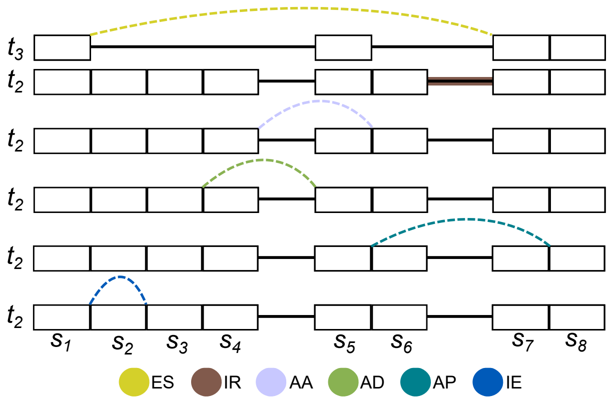

Figure S4: Alternative illustration of alternative splicing events with transcripts and subexons taken from Figure 3.

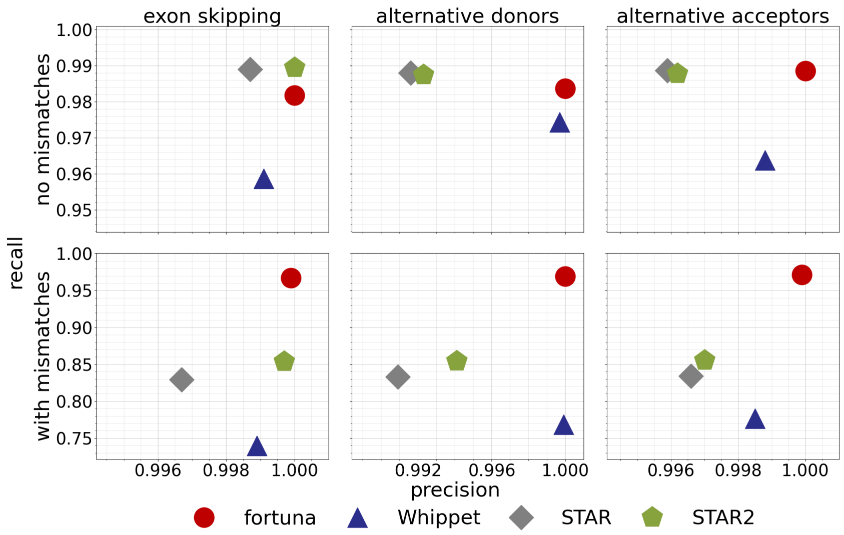

Figure S5: Precision and recall in finding novel junctions between annotated splice sites. Results of fortuna, Whippet, STAR and STAR with two-pass mode (STAR2) are shown for the simulated dataset with 100bp reads. Reads were split into error-free reads (upper row) and reads containing mismatches (bottom row). Results are stratified by event type (columns).

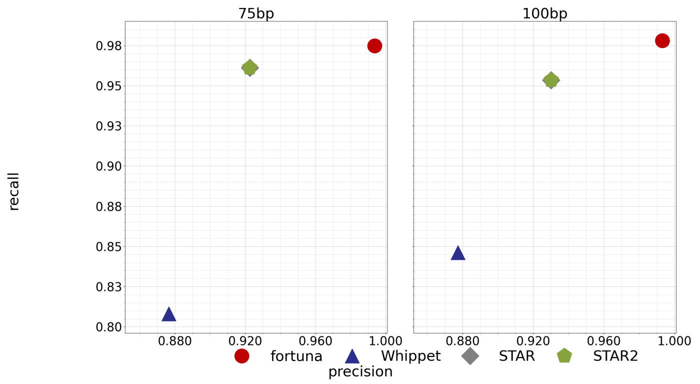

Figure S6: Precision and recall in finding annotated junctions. Results of fortuna, Whippet, STAR and STAR with two-pass mode (STAR2) are shown for the simulated dataset with 75bp and 100bp reads.

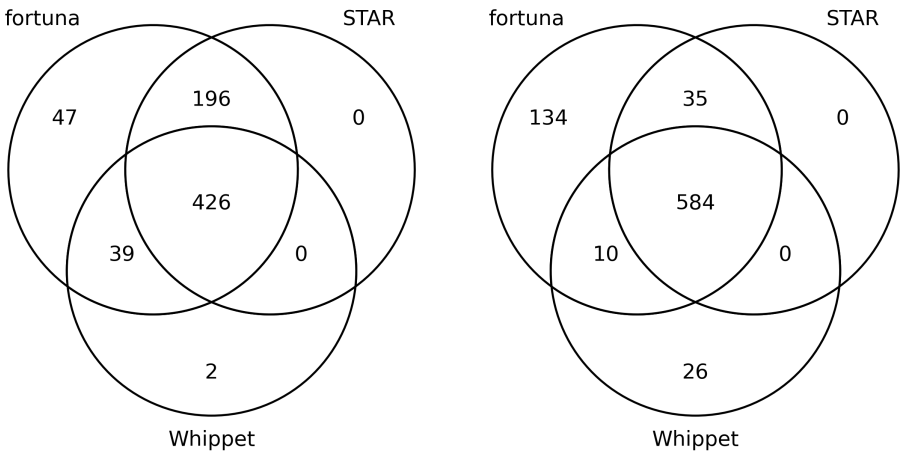

Figure S7: Venn diagram showing the overlap of reads found by each method to support a novel exon skipping event in ASD samples 20 (left) and 36 (right).

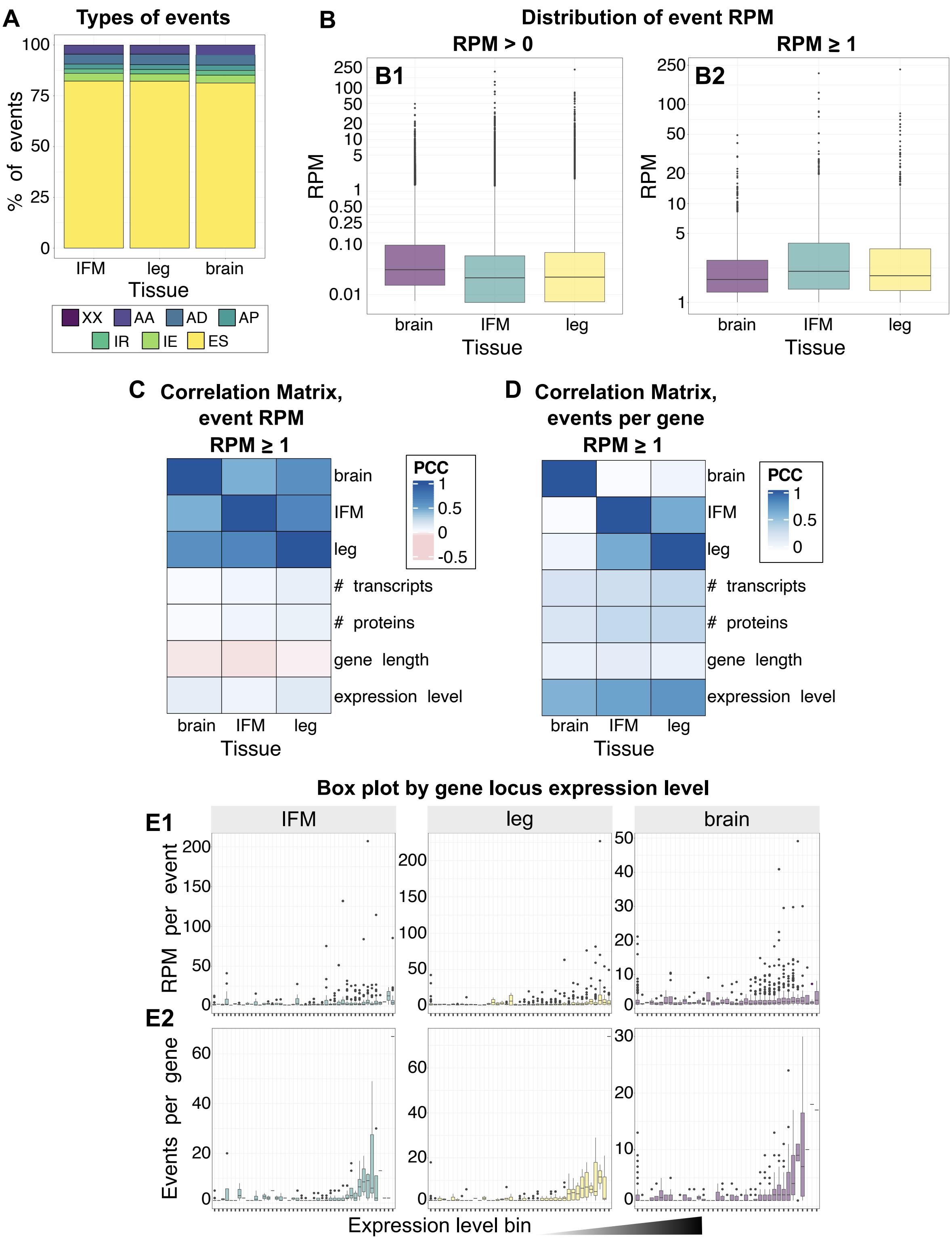

Figure S8: Caption given on the next page.

Figure S8: Event type and correlation between identified events in *Drosophila* tissues. A) Bar plot of the percent of events (RPM 0) classified as ES (yellow), IE (light green), IR (green), AP (cyan), AD (blue), AA (light purple) or XX (purple). B) Box plots showing the distribution of RPM values with RPM 0 (B1) and RPM 1 (B2) for novel events in IFM, leg and brain. C) Correlation plot for filtered events, RPM 1 showing Pearsons correlation coefficient values between tissues and between RPM values and gene length, locus complexity (annotated number of transcripts and proteins) and locus expression level based on DESeq2 normalized read count values. D) Plot of the Pearsons correlation coefficient of events per gene (at filter level RPM 1) between tissues and with gene length, locus complexity and locus expression level based on DESeq2 normalized read count values. E) Box plots of the RPM per event (E1) or the number of events per gene (E2) at a given gene locus expression level. DESeq2 normalized read count values are grouped into 68 bins on the X-axis. Y-axis values are from filtered events with RPM 1 for IFM, leg and brain.

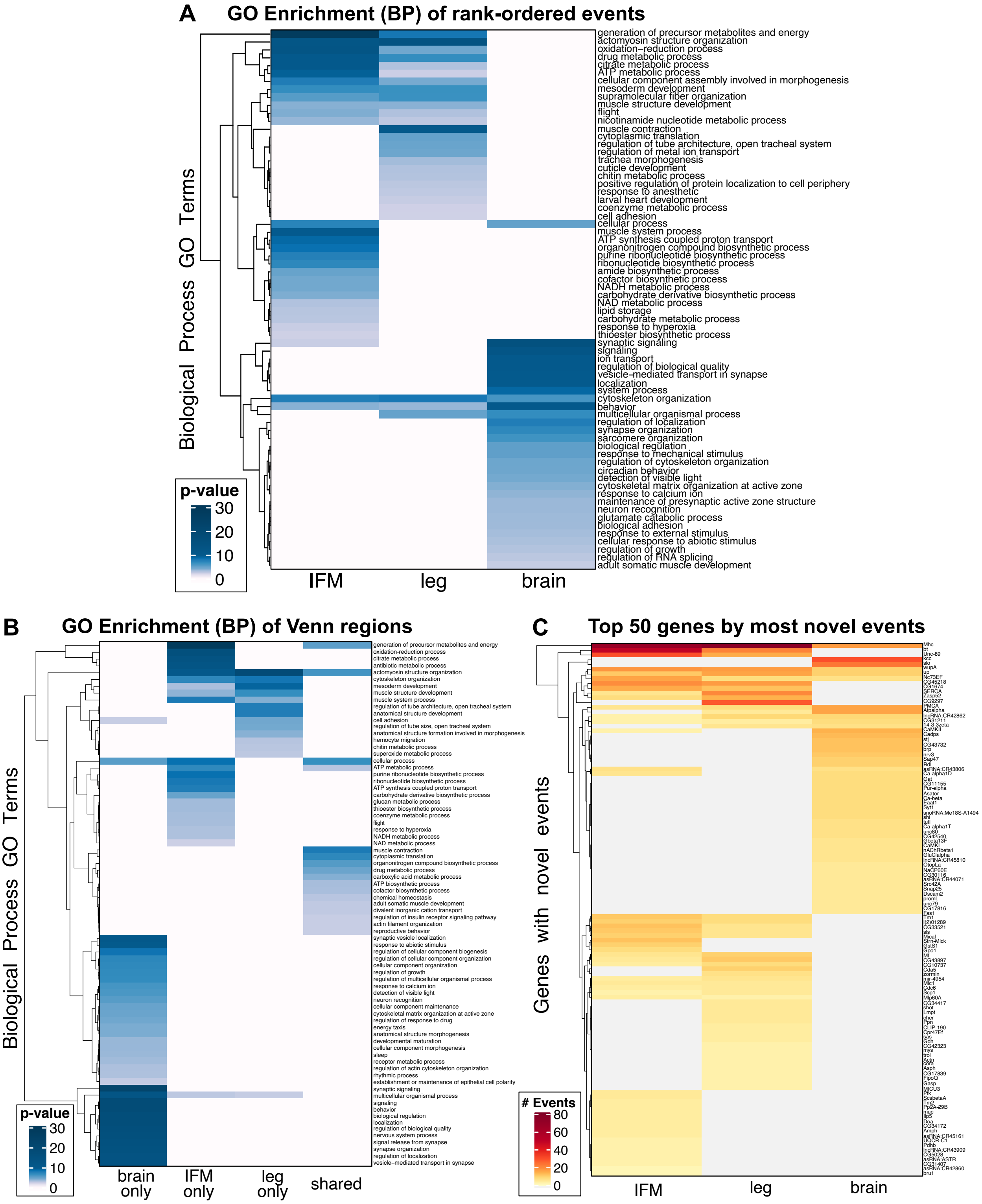

Figure S9: Caption given on the next page.

Figure S9: Novel AS events identified by fortuna are in diverse, tissue-specific genes. A) Clustering and heatmap of enrichment p-values for biological process (BP) gene ontology (GO) terms for novel events. The gene list was rank-ordered by event RPM. B) Clustering and heatmap of enrichment p-values for biological process GO terms for genes from Venn Diagram regions in (Figure 9 C) that are brain, IFM or leg specific, or shared between all tissues. C) Clustering and heatmap of number of novel events per gene from the top 50 genes with the most novel events in each tissue.

	Precision			Recall			F1		
	es	ad	aa	es	ad	aa	es	ad	aa
**75bp**	**no errors**								
fortuna	1.0000	1.0000	1.0000	0.9919	0.9922	0.9962	0.9959	0.9961	0.9981
Whippet	0.9987	0.9993	0.9984	0.9671	0.9815	0.9692	0.9826	0.9903	0.9835
STAR	0.9972	0.9897	0.9960	0.9954	0.9965	0.9958	0.9963	0.9931	0.9959
STAR2	0.9996	0.9925	0.9965	0.9959	0.9962	0.9953	0.9978	0.9943	0.9959
ASGAL	0.9990	0.9898	0.9940	0.8993	0.8019	0.8059	0.9465	0.8860	0.8901
	**with errors**								
fortuna	0.9998	0.9999	1.0000	0.9322	0.9273	0.9321	0.9648	0.9622	0.9649
Whippet	0.9981	0.9992	0.9985	0.6793	0.7163	0.7093	0.8084	0.8344	0.8294
STAR	0.9941	0.9903	0.9968	0.8065	0.8014	0.8067	0.8905	0.8859	0.8917
STAR2	0.9991	0.9956	0.9980	0.8474	0.8425	0.8464	0.9171	0.9127	0.9159
ASGAL	0.9981	0.9939	0.9966	0.4300	0.4017	0.4076	0.6010	0.5721	0.5785
	**all reads**								
fortuna	1.0000	1.0000	1.0000	0.9774	0.9766	0.9804	0.9885	0.9881	0.9901
Whippet	0.9986	0.9993	0.9984	0.8969	0.9176	0.9051	0.9450	0.9567	0.9494
STAR	0.9966	0.9898	0.9962	0.9494	0.9494	0.9492	0.9724	0.9692	0.9721
STAR2	0.9995	0.9931	0.9969	0.9597	0.9590	0.9586	0.9792	0.9758	0.9773
ASGAL	0.9989	0.9904	0.9943	0.7849	0.7051	0.7075	0.8790	0.8237	0.8267
**100bp**	**no errors**								
fortuna	1.0000	1.0000	1.0000	0.9818	0.9837	0.9886	0.9908	0.9918	0.9942
Whippet	0.9991	0.9997	0.9988	0.9587	0.9744	0.9638	0.9784	0.9869	0.9810
STAR	0.9987	0.9916	0.9959	0.9890	0.9880	0.9887	0.9939	0.9898	0.9923
STAR2	1.0000	0.9923	0.9962	0.9895	0.9875	0.9878	0.9947	0.9899	0.9920
ASGAL	0.9996	0.9888	0.9930	0.8479	0.6709	0.6870	0.9175	0.7994	0.8122
	**with errors**								
fortuna	0.9999	1.0000	0.9999	0.9673	0.9692	0.9716	0.9834	0.9844	0.9856
Whippet	0.9989	0.9999	0.9985	0.7395	0.7685	0.7764	0.8498	0.8691	0.8735
STAR	0.9967	0.9909	0.9966	0.8293	0.8328	0.8343	0.9053	0.9050	0.9082
STAR2	0.9997	0.9941	0.9970	0.8536	0.8544	0.8553	0.9209	0.9189	0.9207
ASGAL	0.9988	0.9947	0.9951	0.4130	0.3518	0.3643	0.5844	0.5198	0.5333
	**all reads**								
fortuna	1.0000	1.0000	1.0000	0.9772	0.9791	0.9832	0.9884	0.9894	0.9915
Whippet	0.9990	0.9998	0.9987	0.8889	0.9086	0.9042	0.9408	0.9520	0.9491
STAR	0.9982	0.9914	0.9961	0.9383	0.9384	0.9395	0.9673	0.9641	0.9670
STAR2	0.9999	0.9928	0.9964	0.9462	0.9449	0.9456	0.9723	0.9683	0.9704
ASGAL	0.9995	0.9899	0.9934	0.7095	0.5685	0.5842	0.8299	0.7222	0.7357

Table S1: Precision, recall, and F1 score achieved by fortuna, Whippet, STAR and STAR run in two-pass mode (STAR2), and ASGAL on the two simulated datasets.

	75bp		100bp	
	Precision	Recall	Precision	Recall
fortuna	0.9938	0.9748	0.9932	0.9780
whippet	0.8765	0.8081	0.8774	0.8462
STAR	0.9226	0.9612	0.9301	0.9534
STAR2	0.9225	0.9612	0.9301	0.9534

Table S2: Precision and recall achieved by fortuna, Whippet, STAR and STAR run in two-pass mode (STAR2) on the two simulated datasets with regards to annotated splice junctions.

Sample #	SpliceAI	fortuna	STAR	Whippet	$F\setminus(S\cup W)$	$W\setminus(S\cup F)$	$(F\cap W)\setminus S$	$(F\cap S)\setminus W$	$F\cap W\cap S$
29	45	107	99	96	8	0	3	0	96
12	1	2	2	2	0	0	0	0	2
36	483	763	619	620	134	26	35	10	584
26	1	1	1	1	0	0	0	0	1
20	536	708	622	467	47	2	196	39	426
4	914	1247	1084	1093	130	5	29	33	1055
30	120	971	812	890	72	8	17	87	795

Table S3: Number of reads supporting exon skipping events in 7 samples found in the original publication and by methods fortuna (F), STAR (S) and Whippet (W), as well as the overlap between read sets. Sets $S\setminus(F\cup W)$ and $(W\cap S)\setminus F$ were empty in all samples.

Sample #	11	27	25	9	1	31	7	15
SpliceAI	15	9	6	12	4	12	67	3
fortuna and STAR	14	18	9	19	4	10	110	2

Table S4: Number of reads supporting novel donor and acceptor sites found in the original publication (SpliceAI) and by methods fortuna and STAR. We excluded sample 34, as the splice site in this sample is contained in the RefSeq annotation used in our experiments. Whippet cannot identify novel splice sites unless provided with the mappings by a spliced aligner such as STAR on the entire dataset.

Sample #	quant reads	quant junctions	refine reads	refine junctions
20	8012592	229381	693072	4543
36	9804320	303866	436905	4354
4	4518762	214426	614056	4968
12	5919053	211468	430257	4245
26	7355204	332899	362112	7366
28	5087986	169394	416558	3548
29	4315622	254294	423696	5268
30	5502400	246792	475815	5373

Table S5: Number of novel junctions with annotated splice sites identified by fortuna `--quant` and `--refine` along with the number of reads supporting those junctions.

Sample size	fortuna	fortuna 4t	Whippet	SplAdder pipeline	sigcount pipeline
10	7m6.68s	2m25.77s	12m2.28s	129m34.88s	35m12.35s
20	10m53.11	4m2.31s	30m19.6s	221m20.53s	68m45.44s
30	16m5.68s	5m28.48s	37m32.47s	290m55.9s	91m50.05s
40	19m11.69s	7m13.91s	49m4.49s	405m34.64s	138m53.58s
50	25m38.53s	9m9.84s	53m31.75s	450m15.93s	153m25.16s
60	27m44.49s	9m47.69s	66m35s	548m26.09s	181m35.37s
70	30m57.32s	11m6.35s	78m35s	621m52.54s	208m48.05s
80	35m14.2s	13m7.26s	84m30s	703m18.49s	245m47.34s
90	39m4.49s	14m22.92s	99m19s	825m1.14s	270m47.45s
100	44m32.3s	15m6.44s	107m40s	902m13.33s	310m31.68s

Table S6: Total running times of fortuna and competing methods on random samples of ASD sample 29. SplAdder and sigcount pipelines included alignment by STAR (1-pass mode) and sorting and indexing of alignments. Between 10% and 90% of the full data were randomly sampled. All methods used a single thread, except fortuna 4t which used 4 threads. No alignments were output by fortuna and Whippet in this experiment.

Sample size	STAR	samtools	SplAdder	sigcount	STAR2
10	23m41.301s	7m0.910s	98m52.669s	4m30.14s	44m52.35s
20	47m21.577s	12m55.125s	161m3.831s	8m28.74s	86m20s
30	59m21.023s	19m54.648s	211m40.230s	12m34.38s	127m41s
40	91m23.588s	31m3.876s	283m7.180s	16m26.12s	181m10s
50	99m33.978s	33m11.337s	317m30.619s	20m39.85s	227m56s
60	119m0.250s	37m56.228s	391m29.615s	24m38.89s	275m24s
70	136m10.573s	44m15.756s	441m26.212s	28m21.72s	308m0s
80	158m39.954s	54m40.280s	489m58.254s	32m27.11s	364m22s
90	176m31.204s	57m32.648s	590m57.291s	36m43.60s	414m56s
100	205m15.834s	64m3.729s	632m53.767s	41m12.12s	470m16s

Table S7: Running times of the individual components of the SplAdder and sigcount pipelines on random samples of ASD sample 29. STAR was used for alignment, samtools for sorting and indexing of alignments. STAR2 denotes STAR run in 2-pass mode. Between 10% and 90% of the full data were randomly sampled. Additionally, we report the running times for STAR in 2-pass mode.

Sample size	fortuna	fortuna 4t	Whippet
10	14m54.644s	9m52.400s	15m1.094s
20	27m56.382s	15m52.536s	29m58.323s
30	39m44.237s	21m35.771s	45m22.122s
40	51m12.438s	26m55.868s	61m46.507s
50	63m59.135s	32m15.486s	76m21.020s
60	75m27.335s	38m29.587s	91m20.442s
70	85m12.078s	43m24.902s	110m16.551s
80	97m38.243s	48m2.208s	133m28.780s
90	108m26.154s	53m24.441s	141m41.704s
100	123m54.532s	59m40.263s	157m17.852s

Table S8: Running times of fortuna and Whippet when writing alignments to disk. Between 10% and 90% of ASD sample 29 were randomly sampled. fortuna was run once single-threaded and once with 4 threads (fortuna 4t).

	#Fragments	#Nucleotides	Running time	Memory usage
simulated, read length 75bp				
F	3617483	689052987	3m38s	9.54 GB
$F_{1:1}$	4413353	584604244	3m47s	10.31 GB
F_{max}	3366211	1721588224	5m06s	9.58 GB
simulated, read length 100bp				
F	6038072	1444971175	5m24s	11.77GB
$F_{1:1}$	7764323	1125682831	5m20s	14.54 GB
F_{max}	5833818	2959210243	16m11s	11.93GB
ASD sample 29, read length 151bp				
F	18375312	5847332444	36m58s	20.2 GB
$F_{1:1}$	23476032	4346088101	35m11s	39.88 GB
F_{max}	18253996	9311641302	45m32s	20.72GB

Table S9: Comparison of fragment sets F, $F_{1:1}$ and F_{max} as defined in Section 2.3. The number of fragments and the total number of nucleotides across all fragments generated for the two simulated datasets and ASD sample 29 are shown. In addition, running time and memory usage of pseudoalignment by kallisto to the three different fragment sets are shown for the three datasets.

Table S10: Raw and processed data tables for novel splice events in Drosophila IFM, brain and leg. Tab 1: Description of all tables. Tab 2: Events detected by Fortuna in brain at 72h APF. Tab 3: Events detected by Fortuna in IFM at 72h APF. Tab 4: Events detected by Fortuna in whole leg at 72h APF. Tab 5: Genes with novel events in brain. Tab 6: Genes with novel events in IFM. Genes with novel events in leg. Tab 7: Values for Pearsons correlation coefficient used in the plots in (Figure S2 C, D). Tab 8: Identity of genes/events in each region of the Venn Diagram in (Figure 9 C). Tab 9: Full results of GO analysis of genes with events in brain, IFM or leg rank-ordered by highest event RPM. Tab 10: Full results of GO analysis of genes in VennDiagram regions corresponding to IFM-only, leg-only, brain-only or shared by all three tissues.

Tab Name	Description	Data Columns
brain_fortuna	raw fortuna output matched to gene locus via event coordinates for 72h APF dissected brain sample from Drosophila	seqnames = chromosome; start = novel event start coordinate; end = novel event end coordinate; width = distance in bp between start and end; strand = transcript identifier affected by novel event; event_type = type of event based on fortuna event classification; count = number of reads in dataset that contain the novel event; current_symbol = current gene symbol from Flybase; FBgn = Flybase identifier; RPM = count converted to reads per million; unique_exon_base_count = total unique exon bases in gene locus from Flybase; total_exon_base_count = total exon bases from Flybase; RPKM = count converted to reads per kilobase per million bases

IFM_fortuna	raw fortuna output matched to gene locus via event coordinates for 72h APF dissected IFM sample from Drosophila	seqnames = chromosome; start = novel event start coordinate; end = novel event end coordinate; width = distance in bp between start and end; strand = transcript identifier affected by novel event; event_type = type of event based on fortuna event classification; count = number of reads in dataset that contain the novel event; current_symbol = current gene symbol from Flybase; FBgn = Flybase identifier; RPM = count converted to reads per million; unique_exon_base_count = total unique exon bases in gene locus from Flybase; total_exon_base_count = total exon bases from Flybase; RPKM = count converted to reads per kilobase per million bases
leg_fortuna	raw fortuna output matched to gene locus via event coordinates for 72h APF dissected leg sample from Drosophila	seqnames = chromosome; start = novel event start coordinate; end = novel event end coordinate; width = distance in bp between start and end; strand = transcript identifier affected by novel event; event_type = type of event based on fortuna event classification; count = number of reads in dataset that contain the novel event; current_symbol = current gene symbol from Flybase; FBgn = Flybase identifier; RPM = count converted to reads per million; unique_exon_base_count = total unique exon bases in gene locus from Flybase; total_exon_base_count = total exon bases from Flybase; RPKM = count converted to reads per kilobase per million bases
brain_genedata	all genes with novel events in the 72h APF brain sample	FBgn = Flybase identifier; GeneSymbol = current gene symbol from Flybase; count = raw fortuna count of most frequent novel event in that gene; RPM = RPM of most frequent novel event in that gene; transcript_type = designation from Flybase if the transcript is protein coding or an RNA species; gene_type = designation from Flybase if the locus is protein coding or an RNA species; gene_fullname = full name of the gene; annotation_ID = CG number from Flybase; location_max = base pair coordinate of one end of gene locus; location_min = base pair coordiante of other end of gene locus; number_transcripts = number of annotated transcripts in gene locus; number_proteins = number of annotated proteins in gene locus; gene_length = total length in base pairs of gene locus; DESeq2normcts = gene locus expression level, averaged count value of the locus after DESeq2 normalization from two independent biological replicates; eventspergene.F0 = number of novel events observed in the locus without filtering; F0 to F100 = filter columns, if TRUE a gene meets inclusion (ie at least one event in that gene has an RPM) greater than or equal to the specified threshold, if FALSE it does not

IFM_genedata	all genes with novel events in the 72h APF IFM sample	FBgn = Flybase identifier; GeneSymbol = current gene symbol from Flybase; count = raw fortuna count of most frequent novel event in that gene; RPM = RPM of most frequent novel event in that gene; transcript_type = designation from Flybase if the transcript is protein coding or an RNA species; gene_type = designation from Flybase if the locus is protein coding or an RNA species; gene_fullname = full name of the gene; annotation_ID = CG number from Flybase; location_max = base pair coordinate of one end of gene locus; location_min = base pair coordiante of other end of gene locus; number_transcripts = number of annotated transcripts in gene locus; number_proteins = number of annotated proteins in gene locus; gene_length = total length in base pairs of gene locus; DESeq2normcts = gene locus expression level, averaged count value of the locus after DESeq2 normalization from two independent biological replicates; eventspergene.F0 = number of novel events observed in the locus without filtering; F0 to F100 = filter columns, if TRUE a gene meets inclusion (ie at least one event in that gene has an RPM) greater than or equal to the specified threshold, if FALSE it does not
leg_genedata	all genes with novel events in the 72h APF leg sample	FBgn = Flybase identifier; GeneSymbol = current gene symbol from Flybase; count = raw fortuna count of most frequent novel event in that gene; RPM = RPM of most frequent novel event in that gene; transcript_type = designation from Flybase if the transcript is protein coding or an RNA species; gene_type = designation from Flybase if the locus is protein coding or an RNA species; gene_fullname = full name of the gene; annotation_ID = CG number from Flybase; location_max = base pair coordinate of one end of gene locus; location_min = base pair coordiante of other end of gene locus; number_transcripts = number of annotated transcripts in gene locus; number_proteins = number of annotated proteins in gene locus; gene_length = total length in base pairs of gene locus; DESeq2normcts = gene locus expression level, averaged count value of the locus after DESeq2 normalization from two independent biological replicates; eventspergene.F0 = number of novel events observed in the locus without filtering; F0 to F100 = filter columns, if TRUE a gene meets inclusion (ie at least one event in that gene has an RPM) greater than or equal to the specified threshold, if FALSE it does not

Pearson's correlation	values of Pearson's correlation coefficient (PCC) plotted in Figure Sx B&D	PCC at threshold levels of RPM greater than or equal to 0, 0.5 and 1 for event RPM and number of events per gene. Correlations were examined between brain, IFM and leg as well as to the number of transcripts and number of proteins annotated in that locus, the length of the gene and the locus expression level (DESeq2 normalized counts value)
VennDiagram	data table of genes/events included in each region of the Venn Diagram in Figure xD and for doing the GO enrichments for the ifm only vs leg only vs brain only vs shared gene sets	tables lists gene/event membership for Venn Diagram regions at an RPM threshold greater than or equal to 1. The total counts displayed for each region are also noted. A1 = IFM only; a2 = IFM + leg; a3 = leg only; a4 = IFM + brain; a5 = shared in all 3 tissues; a6 = brain + leg; a7 = brain only
GO_rankordered	full results (after rrvgo reduction) for biological process (BP), cellular component (CC) and molecular function (MF) gene ontology terms of gene lists rank-ordered by event RPM	go = GO term ID; parent = parent term from rrvgo (ie reduced term tree); term = GO term description; parentTerm = parent GO term description; ontology = BP, CC or MF which ontology the GO term belongs to; brain = -log10p-value for term enrichment in brain sample; leg = -log10p-value for term enrichment in leg sample; ifm = -log10p-value for term enrichment in IFM sample
GO_Vennregions	full results (after rrvgo reduction) for biological process (BP), cellular component (CC) and molecular function (MF) gene ontology terms of gene lists from VennDiagram regions for genes with IFM-only, leg-only, brain-only and shared events	go = GO term ID; parent = parent term from rrvgo (ie reduced term tree); term = GO term description; parentTerm = parent GO term description; ontology = BP, CC or MF which ontology the GO term belongs to; brainonly = -log10p-value for term enrichment in brainonly genes; legonly = -log10p-value for term enrichment in legonly genes; ifmonly = -log10p-value for term enrichment in IFMonly genes; shared = -log10p-value for term enrichment in genes with events shared in all 3 tissues

2 Supplementary Methods

2.1 Virtual transcriptomes

Definition (Virtual transcriptome T_g^{ap})**.** *Let* $5'(s)$ *and* $3'(s)$ *denote the genomic coordinate of the first and last nucleotide in subexon s, respectively.* T_g^{ap} *contains all transcripts t defined by* $\iota_t = (i_1, \dots, i_m)$ *such that*

(i) no two "touching" exons from different transcripts can be merged: For any two consecutive subexons s_i, s_{i+1} *in t that together define a contiguous region along the genome, i.e.* $5'(s_{i+1}) = 3'(s_i) + 1$*, there exists a transcript* $t' \in T_g$ *with* $i, i+1 \in \iota_{t'}$*,*

(ii) t contains only annotated donor and acceptor sites, i.e. for any two subexons $s_{i_j}, s_{i_{j+1}}$ *that bound non-empty intron* $[3'(s_i)+1, 5'(s_{i+1})-1]$*, there exist transcripts* $t_1, t_2 \in T_g$ *such that* s_{i_j} *defines a splice donor in* t_1*, and* $s_{i_{j+1}}$ *defines a splice acceptor in* t_2*,*

(iii) t contains only annotated TSS and TES, i.e. s_{i_1} *and* s_{i_m} *are annotated transcription start and end sites, respectively.*

2.2 Fragments generated by fortuna

The leftmost nucleotide $5'_l(\iota)$ a read of length l can cover in s_1

$$5'_l(\iota) = \max\left\{5'(s_{i_1}), 3'(s_{i_1}) - (l - 1 - \sum_{j=2}^{m-1} |s_{i_j}|) + 1\right\} \tag{1}$$

and the rightmost nucleotide in s_n it can cover is

$$3'_l(\iota) = \min\left\{5'(s_{i_m}) + (l - 1 - \sum_{j=2}^{m-1} |s_{i_j}|) - 1, 3'(s_{i_m})\right\} \tag{2}$$

Formally, we call a feasible signature $\iota = (i_1, \dots, i_m)$ maximal if no feasible signature ι' exists with $\iota \subseteq_c \iota'$. Then the following set of fragments contains the same set of contiguous subsequences of length l as $F_{1:1}$.

$$F_{max} := \{[s_{i_1}] \cdot \ \dots \ \cdot [s_{i_m}] \mid \iota \text{ is maximal feasible signature given } T, l\} \tag{3}$$

fortuna trims sequences at the end of fragments to at most $l-1$ bases in the first and last subexons:

$$\begin{aligned} F_{trim} := \{ & [\max\{5'(s_{i_1}), 3'(s_{i_1}) - l + 2\}, 3'(s_{i_1})] \\ & \cdot [s_{i_2}] \ \cdot \ \dots \ \cdot \ [s_{i_{m-1}}] \\ & \cdot [5'(s_{i_m}), \min\{5'(s_{i_m}) + l - 2, 3'(s_{i_m})\}] \\ & \mid \iota \text{ is maximal feasbile signature with } m \geq 2\}. \end{aligned}$$

Note that no subexon contributes more than $l-1$ bases to the sequence of any of the fragments in F_{trim}. Finally, in fortuna we generate the set of fragments

$$F = F_{trim} \cup \{[s_{i_1}] \mid \iota \text{ is a feasible signature with } m = 1\}. \tag{4}$$

Calculation of F_{max}

In the following, we describe the algorithms used to compute fragment sets $F_{1:1}$ and F_{max}. For every gene, we create a directed graph with subexons as its nodes, and an edge from subexon s_i to subexon s_j if and only if $5'(s_i) < 5'(s_j)$. Transcript fragments are generated by traversing the graph in a depth-first search (DFS) manner starting from every subexon in increasing order of their coordinates. Edges that would imply infeasible signatures according to properties (f_1) or (f_3) in Definition 2 are pruned. All other enumerated paths that additionally satisfy $(f2)$ together form fragment set $F_{1:1}$. Note that the directed graph is never constructed explicitly. Properties (f_1)-(f_3) implicitly define its structure.

To retain only fragments in F_{max} that correspond to signatures that are not contained in other feasible signatures, every signature generated by the above procedure is added to a suffix trie data structure. Since we start DFS from every subexon from smaller to larger coordinate, signatures that contain another signature will be generated before their contained signatures. Trying to add such a contained signature later to the suffix trie will thus fail to create a new node in the trie and will thus be discarded.

Proof of Theorem 1.

Statement (1) follows directly from property f_1 in Main Paper Definition 2. Let read r of length l cover all subexons in some signature $s(f)$. By Main Paper Definition 2, f satisfies f_1-f_3. If f is maximal the claim follows. Otherwise, there must exist $f' \in F$ such that $s(f) \subseteq_c s(f')$. Then, r can be sampled from f'. ■

2.3 Incorporation of novel splice sites

fortuna optionally (`--refine`) incorporates novel splice sites found by STAR or any other spliced aligner from reads that fortuna was not able to (pseudo)align to transcripts in T^{as} or T^{ap}. This potentially also includes novel junctions between annotated splice sites that are not contained in T^{as} or T^{ap}. It incorporates spliced alignments by splitting every subexon at every novel splice site it contains as follows. Let the spliced alignment of a read be represented by a sequence of n contiguous (ungapped) genomic intervals $((a_j, b_j))_{j=1}^{n}$. Let ι_j denote the sequence of indices of subexons that overlap the jth interval, i.e. for each s_i with $i \in \iota_j$, $(5'(s_i), 3'(s_i)) \cap (a_j, b_j) \neq \emptyset$. For each $j = 1, \ldots, n$, we subdivide subexons as follows.

- If $j = 1$, let s_i with $i \in \iota_j$ be the subexon that contains b_1. We partition s_i into subexons $(5'(s_i), b_1)$ and $(b_1 + 1, 3'(s_i))$.
- If $j = m$, let s_i with $i \in \iota_j$ be the subexon that contains a_m. We partition s_i into subexons $(5'(s_i), a_m - 1)$ and $(a_m, 3'(s_i))$.
- If $1 < j < n$, let subexon s_i with $i \in \iota_j$ contain a_j and $s_{i'}$, $i' \in \iota_j$ contain b_j. If $i = i'$, we partition it into three subexons $(5'(s_i), a_j - 1)$, (a_j, b_j) and $(b_j + 1, 3'(s_i))$. Otherwise, we partition s_i into $(5'(s_i), a_j - 1)$, $(a_j, 3'(s_i))$ and $s_{i'}$ into $(5'(s_{i'}), b_j - 1)$, $(b_j, 3'(s_{i'}))$.

Note that a_1 and b_m do not represent splice sites in the first two cases. In addition, we split counts into new subclasses if reads overlap different stretches of subexons that partition the first and the last subexon of its original mapping signature. To allow for novel transcribed introns or intronic segments, we fill all intronic gaps with subexons before applying the subdivision of subexons. That is, we add one

subexon for each intronic sequence that is not part of any annotated transcript, e.g. one subexon for the intron between subexons s_1 and s_2 and one for the intron between s_3 and s_4 in Figure 2.

2.4 Alternative splicing events

Consistent with previous notation, let $5'(e)$ and $3'(e)$ denote the genomic coordinate of the first and last nucleotide in exon e, and let the novel intron be enclosed by subexons s_{i_j} and $s_{i_{j+1}}$ as part of signature $\iota_r = (i_1, \ldots, i_m)$ of read r.

Definition (exon skipping). *If there exist exons e_1, e_2, e_3 in t in strand order such that $\iota_{e_1} \cap (i_1, \ldots, i_j) \neq \emptyset$, $\iota_{e_3} \cap (i_{j+1}, \ldots, i_m) \neq \emptyset$ and $\iota_{e_2} \cap \iota_r = \emptyset$, the novel intron implies an* exon skipping *with respect to t. In its classical form, $3'(e_1) = 3'(s_{i_j})$ and $5'(e_3) = 5'(s_{i_{j+1}})$.*

Note that the previous definition specifies the skipping of at least one exon but also includes events in which multiple exons are skipped.

Definition (alternative donor). *If there exist consecutive exons e_1, e_2 in t such that $(i_1, \ldots, i_j) \cap \iota_{e_1} \neq \emptyset$ and $5'(s_{i_{j+1}}) = 5'(e_2)$, the novel intron implies an* alternative donor *with respect to t.*

Definition (alternative acceptor). *If there exist consecutive exons e_1, e_2 in t such that $(i_{j+1}, \ldots, i_m) \cap \iota_{e_2} \neq \emptyset$ and $3'(s_{i_j}) = 3'(e_1)$, the novel intron implies an* alternative acceptor *with respect to t.*

Definition (alternative donor-acceptor). *If there exist consecutive exons e_1, e_2 in t such that $(i_1, \ldots, i_j) \cap \iota_{e_1} \neq \emptyset$, $(i_{j+1}, \ldots, i_m) \cap \iota_{e_2} \neq \emptyset$ and $3'(s_{i_j}) \neq 3'(e_1)$, $5'(s_{i_{j+1}}) \neq 5'(e_2)$, the novel intron implies an* alterantive donor-acceptor pair *wrt with respect to t.*

Definition (intron in exon). *If there exists an exon e in t such that $i_j \in \iota_e$ and $i_{j+1} \in \iota_e$, the intron implies a novel* intron in exon *with respect to t.*

Novel transcribed introns are not part of any transcript guessed by fortuna. We therefore employ mappings by a spliced aligner to infer novel intron retentions. To verify no additional splicing occurred within an intron, foruna first refines subexons and intronic gaps as described in Supplementary Section 2.3 and then requires that the intron was not subdivided due to the presence of spliced reads.

3 Drosophila analysis

3.1 Bioinformatic processing of fortuna output

fortuna output was processed in R. Identified events were matched to corresponding gene locus by coordinates using the GenomicRanges package. Biological duplicates and paired reads files were combined to generate a single file for each sample. Raw counts were converted to RPM to enable comparison between libraries. Novel splice acceptors (SA) and splice donors (SD) were identified in comparison to the ENSEMBL transcriptome annotation. Gene ontology (GO) enrichments for rank-ordered lists were performed using GOrilla[1], and lists of terms were simplified using rrvgo. For correlation plots, transcript number, protein number and gene length of all annotated loci was obtained from Flybase. DESeq2 normalized counts were used for locus expression level values. Pearsons correlation coefficient

[1] `http://cbl-gorilla.cs.technion.ac.il/`

was calculated either between event RPM values or the number of events per gene. RPM per event and the number of events per gene were additionally plotted in sequential bins by gene locus expression level. Packages employed include table, VennDiagram, ggplot2, viridis, plyr, dplyr, reshape2, ComplexHeatmap, circlize, RColorBrewer, rrvgo and org.Dm.eg.db. Processed data tables are available in the supplemental materials.

3.2 mRNA-Seq on Drosophila tissues

IFM, brain and leg were dissected from w^{1118} pupae at 72h after puparium formation (APF) and RNA prepared as described previously [1]. Library preparation and Illumina sequencing were performed by LC Sciences (Houston, TX). Libraries were sequenced as stranded, paired, 100 bp length to a depth >70 million reads. Data is available from GEO under accession number (submitted, not yet available). Sequenced libraries were mapped to the Drosophila genome (BDGP6.22) using STAR or processed with fortuna.

3.3 RT-PCR on Drosophila IFM

IFM was dissected from 1d adult w^{1118} flies, and RNA isolated using TRIzol (Invitrogen) following the manufacturer protocol. Reverse transcription and cDNA synthesis was performed with the Luna Universal One-Step RT-qPCR Kit (NEB). Events were PCR amplified with the following primers: Bru1Ex10FOR- TCGAGTTTTGCCAATACGTCGGG, Bru1Ex18REV- CCTCTACTCGATGTGTCCGTTTAACCTT (annotated isoform 1811 bp, novel isoform 1371 bp), btEx34FOR- ATGATGACGGTGGTTGTGAA, btEx38REV- TACCAGTGGCGGTATCCATT (annotated isoform 2733 bp, novel isoform 933 bp), upEx2FOR- AGGTCGTCGAGGAGGAAGAT, upEx6REV- CCTTCTTCAAGGCTTTGTGC (annotated isoforms 566 bp, novel isoform 406 bp). PCR products were verified based on expected length and Sanger sequencing.

References

[1] S. Kao, E. Nikonova, K. Ravichandran, and M. L. Spletter. Dissection of drosophila melanogaster flight muscles for omics approaches. *JoVE Journal*, 152:e60309, 2019.
